# Selective and catalytic carbon dioxide and heteroallene activation mediated by cerium N-heterocyclic carbene complexes†
†Electronic supplementary information (ESI) available. CCDC 1856101–1856106, 1868204–1868209. For ESI and crystallographic data in CIF or other electronic format see DOI: 10.1039/c8sc03312a


**DOI:** 10.1039/c8sc03312a

**Published:** 2018-09-10

**Authors:** Polly L. Arnold, Ryan W. F. Kerr, Catherine Weetman, Scott R. Docherty, Julia Rieb, Faye L. Cruickshank, Kai Wang, Christian Jandl, Max W. McMullon, Alexander Pöthig, Fritz E. Kühn, Andrew D. Smith

**Affiliations:** a EaStCHEM School of Chemistry , University of Edinburgh , The King's Buildings , Edinburgh , EH9 3FJ , UK . Email: Polly.Arnold@ed.ac.uk; b EaStCHEM School of Chemistry , University of St. Andrews , North Haugh, St. Andrews , KY16 9ST , UK, E-mail: ads10@st-andrews.ac.uk; c Molecular Catalysis, Faculty of Chemistry and Catalysis Research Center , Technical University Munich , Lichtenbergstr. 4 , 85748 Garching bei München , Germany

## Abstract

A series of rare earth complexes of the form Ln(L^R^)_3_ supported by bidentate *ortho*-aryloxide–NHC ligands are reported (L^R^ = 2-O-3,5-^t^Bu_2_-C_6_H_2_(1-C{N(CH)_2_N(R)})); R = ^i^Pr, ^t^Bu, Mes; Ln = Ce, Sm, Eu). The cerium complexes cleanly and quantitatively insert carbon dioxide exclusively into all three cerium carbene bonds, forming Ce(L^R^·CO_2_)_3_. The insertion is reversible only for the mesityl-substituted complex Ce(L^Mes^)_3_. Analysis of the capacity of Ce(L^R^)_3_ to insert a range of heteroallenes that are isoelectronic with CO_2_ reveals the solvent and ligand size dependence of the selectivity. This is important because only the complexes capable of reversible CO_2_-insertion are competent catalysts for catalytic conversions of CO_2_. Preliminary studies show that only Ce(L^Mes^·CO_2_)_3_ catalyses the formation of propylene carbonate from propylene oxide under 1 atm of CO_2_ pressure. The mono-ligand complexes can be isolated from reactions using LiCe(N^i^Pr_2_)_4_ as a starting material; LiBr adducts [Ce(L^R^)(N^i^Pr_2_)Br·LiBr(THF)]_2_ (R = Me, ^i^Pr) are reported, along with a hexanuclear N-heterocyclic dicarbene [Li_2_Ce_3_(OArC^Me^–H)_3_(N^i^Pr_2_)_5_(THF)_2_]_2_ by-product. The analogous *para*-aryloxide–NHC proligand (*p*-L^Mes^ = 4-O-2,6-^t^Bu_2_-C_6_H_2_(1-C{N(CH)_2_NMes}))) has been made for comparison, but the rare earth tris-ligand complexes Ln(*p*-L^Mes^)_3_(THF)_2_ (Ln = Y, Ce) are too reactive for straightforward Lewis pair separated chemistry to be usefully carried out.

## Abstract 2

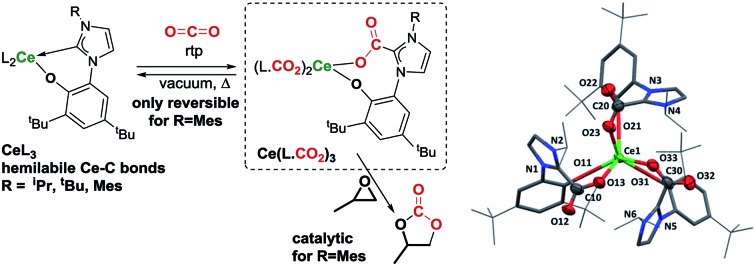
A series of rare earth complexes of the form Ln(L^R^)_3_ supported by bidentate *ortho*-aryloxide–NHC ligands are reported (L^R^ = O(*o*-C_6_H_2_–^t^Bu_2_-2,6-CN(C_2_H_2_)NR); R = ^i^Pr, ^t^Bu, Mes; Ln = Ce, Sm, Eu).

## 

Carbon dioxide can be a useful and renewable C1 building block in the fine and bulk chemical industries due to its natural abundance and reactivity,^1^,^2^ and can provide carboxylic acids, esters and (cyclic) carbonates.^3^ Isoelectronic isocyanates and isothiocyanates are also valuable electrophilic elementary reagents used in polymerization and cyclisations,^4^–^6^ and thus chemistry which utilizes heteroallenes is of great interest.

Lewis basic N-heterocyclic carbenes (NHCs) are known to react with carbon dioxide, isocyanates and isothiocyanates as nucleophiles to form imidazolium carboxylates,^7^,^8^ imidazolium amidates^9^ and imidazolium carbimidothioates^10^ respectively **A** (Chart 1). While imidazolium carboxylates can successfully catalyse carbamate formation,^11^ NHCs react as organocatalysts with isocyanates to form cyclic ureas **B** through an azolium amidate intermediate.^9^

**Chart 1:**
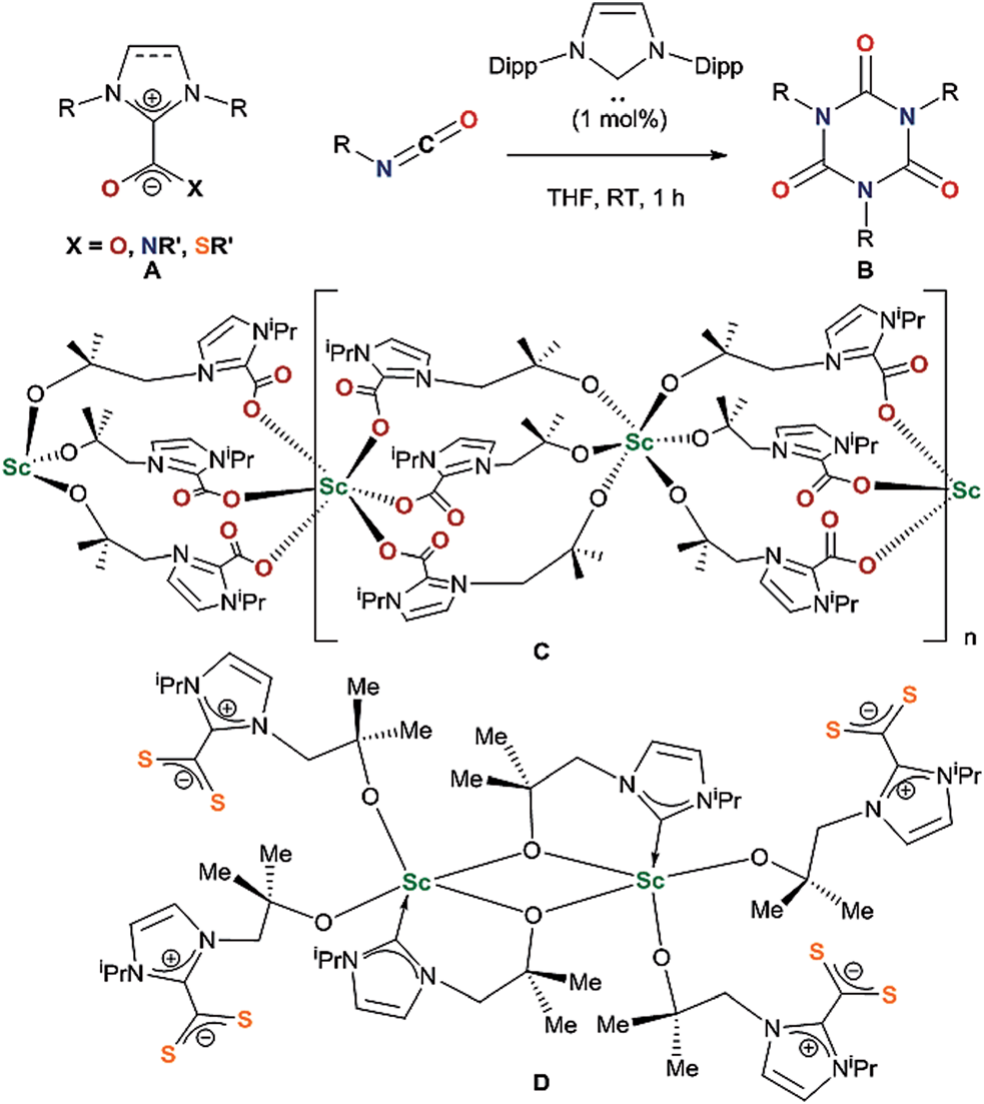


Since the first reported isolation of lanthanide–NHC complexes in 1994,^12^,^13^ it has been shown that Lewis acidic rare-earth cations form hemilabile bonds with soft σ-donating NHCs.^14^,^15^ Between 2006 and 2010, Shen and co-workers published syntheses of aryloxide–NHC lanthanide complexes, however no subsequent reactivity was reported.^16^–^19^ In 2014, we reported the activation of carbon dioxide **C** and carbon disulfide **D** using a scandium alkoxide-NHC complex, achieving frustrated Lewis pair (FLP) like reactivity which resulted in metal–ligand scrambling to form a polymeric –(Sc–NHC–CO_2_)–_*n*_ containing network owing to the flexible alkoxide tether.^20^

Cerium, the most abundant lanthanide has a relatively low toxicity; its trichloride is six times less toxic by ingestion than that of iron,^21^,^22^ and it has many applications in heterogeneous catalysis.^23^ Previously we showed that cerium-silylamido NHC complexes ([Ce(L)(N{SiMe_3_}_2_)_2_] L = bidentate alkoxy-tethered NHC ligand) react with CO_2_ to form an insoluble mixture while the uranium analogue [U(L)(N{SiMe_3_}_2_)_2_] yields an equivalent of isocyanate.^24^ In the latter instance it was not possible to isolate any intermediate that confirmed whether the NHC group was definitively involved in the CO_2_ activation.^25^ Recently, Suresh reported the first mononuclear *N*-carboxylate imidazolium lanthanide compounds, suggesting their potential use as single-molecule magnets.^26^ Here, we demonstrate that cerium (and other rare earth) complexes with aryloxide-tethered NHC ligands can successfully form homoleptic cerium imidazolium carboxylate complexes from CO_2_ insertion into the Ce–C carbene bonds. We show how to control the reversibility for the first time, and use this, and the extent of insertion of CO_2_ or isoelectronic heteroallenes (isocyanates and isothiocyanates) by changing the ligand steric and electronic properties, and by solvent effects. This is important as we show that only the complexes capable of reversible CO_2_-insertion are competent catalysts for the synthesis of cyclic carbonates from CO_2_ and epoxides.

## Results and discussion

### *ortho*-Aryloxide Ln–NHC complex synthesis

**Scheme 1:**
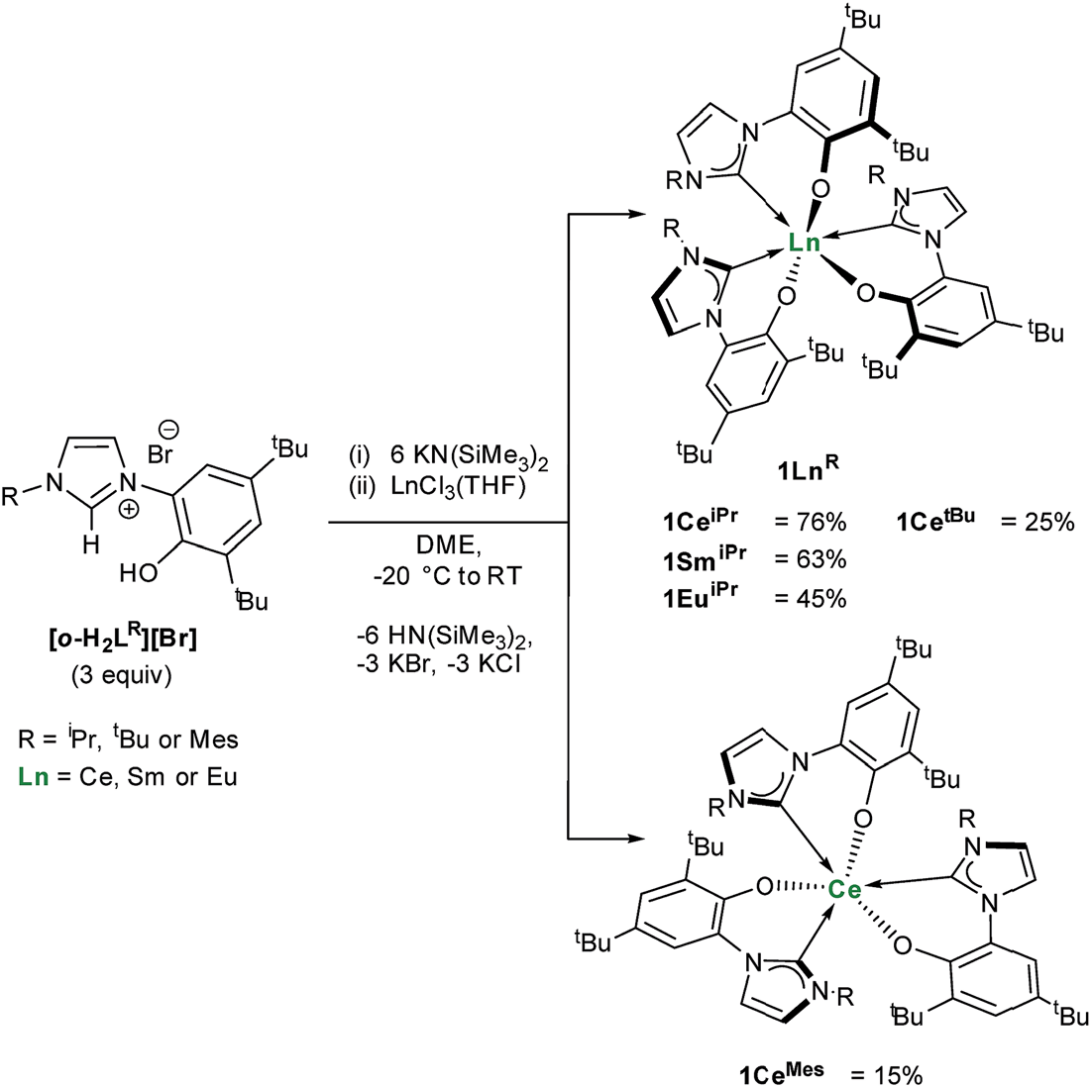
Synthesis of homoleptic lanthanide(iii) complexes **1Ln^R^**.

One objective for synthesizing lanthanide aryloxide tethered–NHC complexes is to combine valuable hemilability within a rigid framework for selective reactivity and we envisioned that varying coordination environments arising from respective alkyl and aryl substituents could give distinctive chemistry. A suspension of an *ortho*-aryloxide NHC proligand,^27^,^28^ **[*o*-H_2_L^R^][Br]** where L^R^ = 2-O-3,5-^t^Bu_2_-C_6_H_2_(1-C{N(CH)_2_N(R)}) and R = ^i^Pr, ^t^Bu and Mes were treated with 6 equivalents of KN(SiMe_3_)_2_ and LnCl_3_(THF)_*n*_ (Ln = Ce, Sm, Eu) in DME to afford bright yellow solutions with colourless precipitates of KCl and KBr (Scheme 1). After work-up, **1Ln^R^** (Ln(L^R^)_3_) can be afforded in moderate to good yields (15–76%), while over 8 g of **1Ce^iPr^** can be isolated in a single reaction.

The ^1^H NMR spectra of all four lanthanide complexes bearing alkyl R groups (^i^Pr or ^t^Bu) contain a complex set of paramagnetic resonances indicating *C*_1_ symmetry and a unique environment for each ligand. In agreement with the ^1^H spectrum of **1Ce^iPr^** the ^13^C{^1^H} NMR spectrum is also complicated, containing three carbene chemical shifts (*δ* = 174.8 ppm, 187.8 ppm, 192.3 ppm), slightly broadened compared to the rest of the spectrum (average fwhm 12 Hz), and shifted compared to similar diamagnetic lanthanide NHC complexes (normal region ≈ 200–238 ppm for Y^III^ and Ce^IV^).^14^,^17^,^19^,^29^,^30^ However in contrast, spectra of **1Ce^Mes^** contain a single set of paramagnetically shifted resonances indicating *C*_3_ symmetry in solution on the ^1^H NMR spectroscopic timescale and ^13^C NMR spectroscopy of **1Ce^Mes^** displays a single carbene resonance at 184.2 ppm.

These ligand orientation differences are rationalized by consideration that three planar mesityl groups pack more easily than the aryloxide/*tert*-butyl groups would, and that the *tert*-butyl/*iso*-propyl steric repulsions are less prescriptive. The *C*_3_-symmetric complex would also be favoured if π-stacking between the mesityl substituent and an adjacent imidazolin-2-ylidine ring is possible. This high degree of steric crowding is used to rationalise the failed synthesis of related but bulkier diisopropylphenyl containing aryloxide-carbene ligands. Reactions aimed at targeting the mono- and bis-alkoxy–NHC analogues using this synthetic method yielded only the tris-ligand complex and unreacted LnCl_3_ while the targeted synthesis of a Sm(ii) analogue results in spontaneous oxidation and isolation of Sm(iii) compound, **1Sm^iPr^** (see ESI†).

**Fig. 1:**
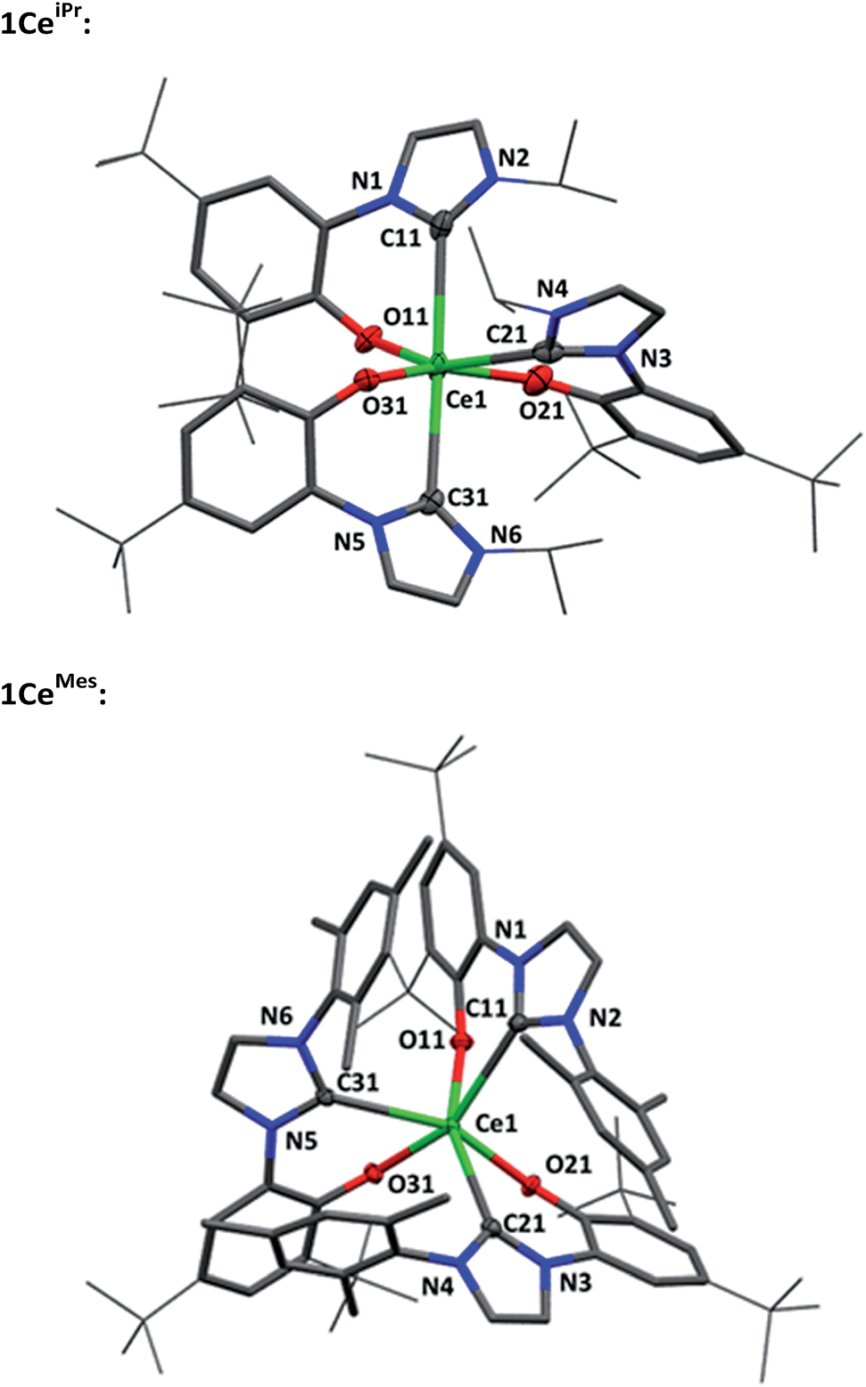
Molecular structures of **1Ce^iPr^** (upper) and **1Ce^Mes^** (lower) with Ce, O and C_carbene_ shown at 50% ellipsoid probability, framework and peripheral carbon atoms drawn capped stick and wireframe respectively, and H and lattice solvent omitted for clarity. Selected distances (Å) and angles (°) for **1Ce^iPr^**: Ce1–C11 2.747(6), Ce1–C21 2.694(6), Ce1–C31 2.785(7), Ce1–O11 2.349(4), Ce1–O21 2.277(4), Ce1–O31 2.283(5), C11–Ce1–C21 88.28(18), C11–Ce1–C31 172.93(18), C21–Ce1–C31 88.53(18), O11–Ce–O21 154.05(15), O11–Ce–O31 107.42(15), O21–Ce–O31 97.45(16), C11–Ce1–O11 68.68(16), C21–Ce1–O21 69.09(18), C31–Ce1–O31 69.66(17); for **1Ce^Mes^**: Ce1–C11 2.823(3), Ce1–C21 2.814(3), Ce1–C31 2.806(3), Ce1–O11 2.266(2), Ce1–O21 2.264(2), Ce1–O31 2.251(2), C11–Ce1–C21 105.49(9), C11–Ce1–C31 100.01(9), C21–Ce1–C31 101.48(9), O11–Ce1–O21 93.66(8), O11–Ce1–O31 96.58(8), O21–Ce1–O31 94.23(8), C11–Ce1–O11 65.85(9), O21–Ce1–O21 66.82(8), O31–Ce1–O31 66.25(9).

Single crystal X-ray analyses show that the *fac*- and *mer*-isomers are retained in the solid state for **1Ce^Mes^** and **1Ce^iPr^** respectively (see Fig. 1). The coordination geometry of cerium in each is a pseudo-octahedral geometry defined by average C–Ce–C bond angles (172.93(18)°/88.41(18)° and 102.33(9)°) and OArCeOAr bond angles (154.05(15)°/102.44(16)° and 94.82(9)°). The average Ce–C bond distances of **1Ce^iPr^** and **1Ce^Mes^** are 2.742(6) Å and 2.814(3) Å with the former within the regular range of a lanthanide–carbene bond. To the best of our knowledge the latter is the longest aryloxide tethered metal–carbene bond and amongst the longest lanthanide–carbene bonds known, consistent with the proposed high degree of hemilability. For **1Ce^Mes^** there is a conceivable offset aromatic donor–acceptor interaction between the electron deficient imidazolin-2-ylidine and the electron rich mesityl with an average centroid distance of 4.36 Å, within the upper limits of face-centred π-stacking.^31^,^32^

### Reactivity of **1Ce^R^** complexes

**Scheme 2:**
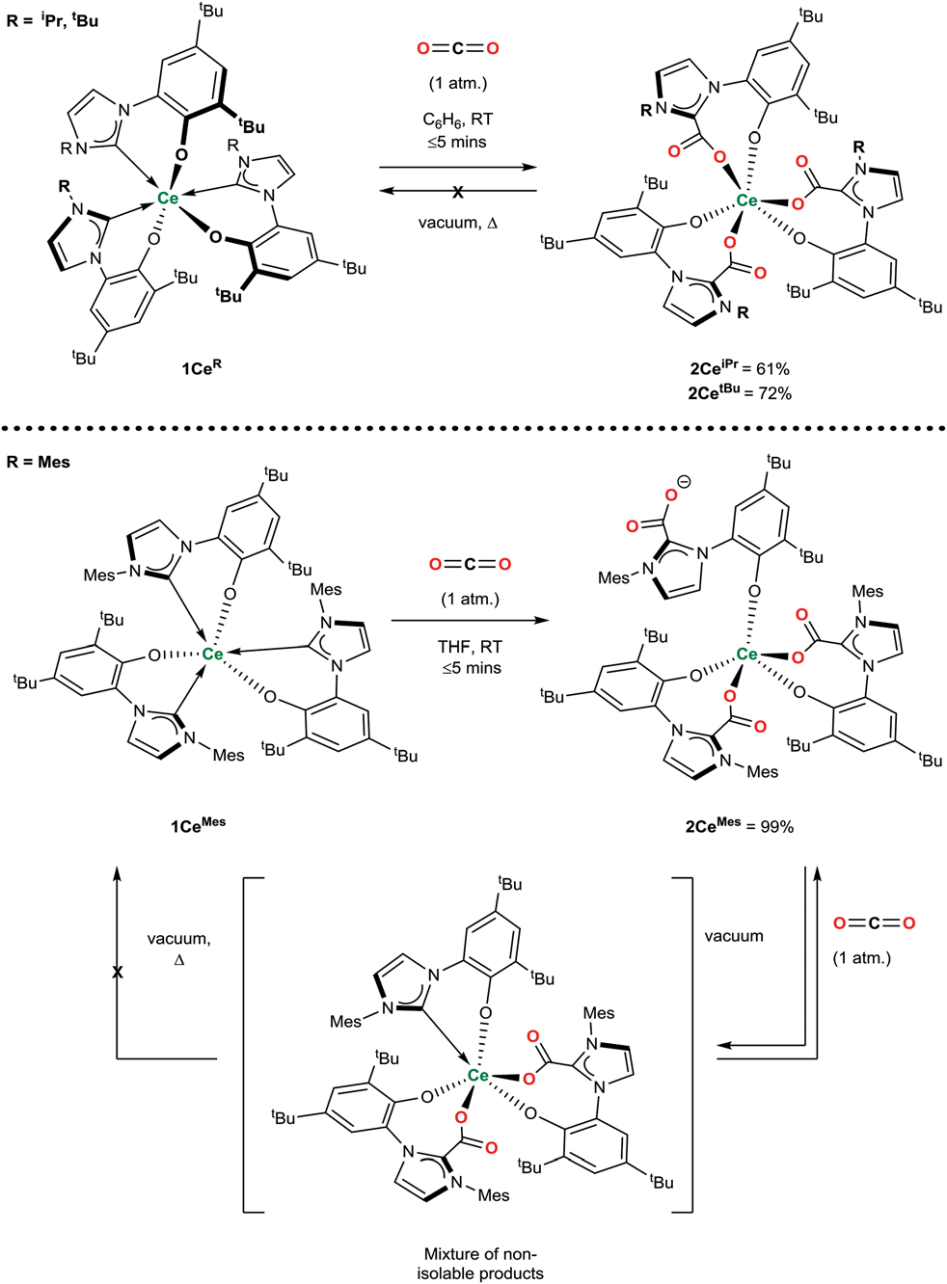
Reactivity of **1Ce^R^** with CO_2_ that forms the triply CO_2_–NHC inserted adducts irreversibly (**2Ce^iPr^** and **2Ce^tBu^**) and reversibly (**2Ce^Mes^**).

Exposure of a solution of **1Ce^R^** to an atmosphere of carbon dioxide results in the instant and quantitative formation of **2Ce^R^** (Ln(L^R^·CO_2_)_3_) as observed by the precipitation of a beige solid (hexanes reaction solvent) or monitoring by ^1^H NMR spectroscopy (benzene reaction solvent), Scheme 2. As anticipated for a complex with a hemilabile metal–NHC bond, the CO_2_ exclusively inserts into the three Ce–C bonds, and pleasingly, and in contrast to the complexes with more flexible, bidentate alkoxide–NHCs, the rest of the molecule remains relatively unperturbed, with no evidence of ligand redistribution between metal centres. Samples of **2Ce^iPr^** and **2Ce^tBu^** held at elevated temperatures under dynamic vacuum (100 °C, 10^–3^ mbar) show no loss of CO_2_. However, a sample of **2Ce^Mes^** shows some loss of CO_2_ under dynamic vacuum (25 to 100 °C, 10^–3^ mbar), that is fully reversible. Solution phase analysis of the material formed shows it to be a complicated mixture that could be oligomeric, but the material is quantitatively converted back to **2Ce^Mes^** upon re-exposure to an atmosphere of CO_2_.

^1^H NMR spectroscopic analysis reveals that the *N*-alkyl functionalised **2Ce^R^** complexes have *C*_3_ symmetry, *i.e.* a *fac*-conformation of the three bidentate ligands. The ^13^C NMR spectra contain diagnostic CO_2_ carbon resonances for **2Ce^iPr^** and **2Ce^tBu^** (*δ* = 173.1 ppm and 173.5 ppm respectively) at significantly higher frequency than known organic NHC·CO_2_ compounds (≈20 ppm)^7^,^8^ as might be anticipated from proximity to the paramagnetic metal center. The FTIR spectrum of **2Ce^iPr^** shows a characteristic absorption at 1666 cm^–1^ (typical range ∼ 1630–1690).^8^,^33^,^34^ The conversion of **1Ce^Mes^** to **2Ce^Mes^** results in a lowering of symmetry from *C*_3_ to *C*_1_ according to room temperature solution spectroscopies. The ^1^H NMR spectrum shows three broadened sets of paramagnetic ligand resonances, and two C–O stretches observable in the FTIR spectrum (1678 and 1715 cm^–1^). We suggest that due to steric hindrance of three mesityl groups that one of the imidazolium carboxylate units is non-coordinating in solution.

**Fig. 2:**
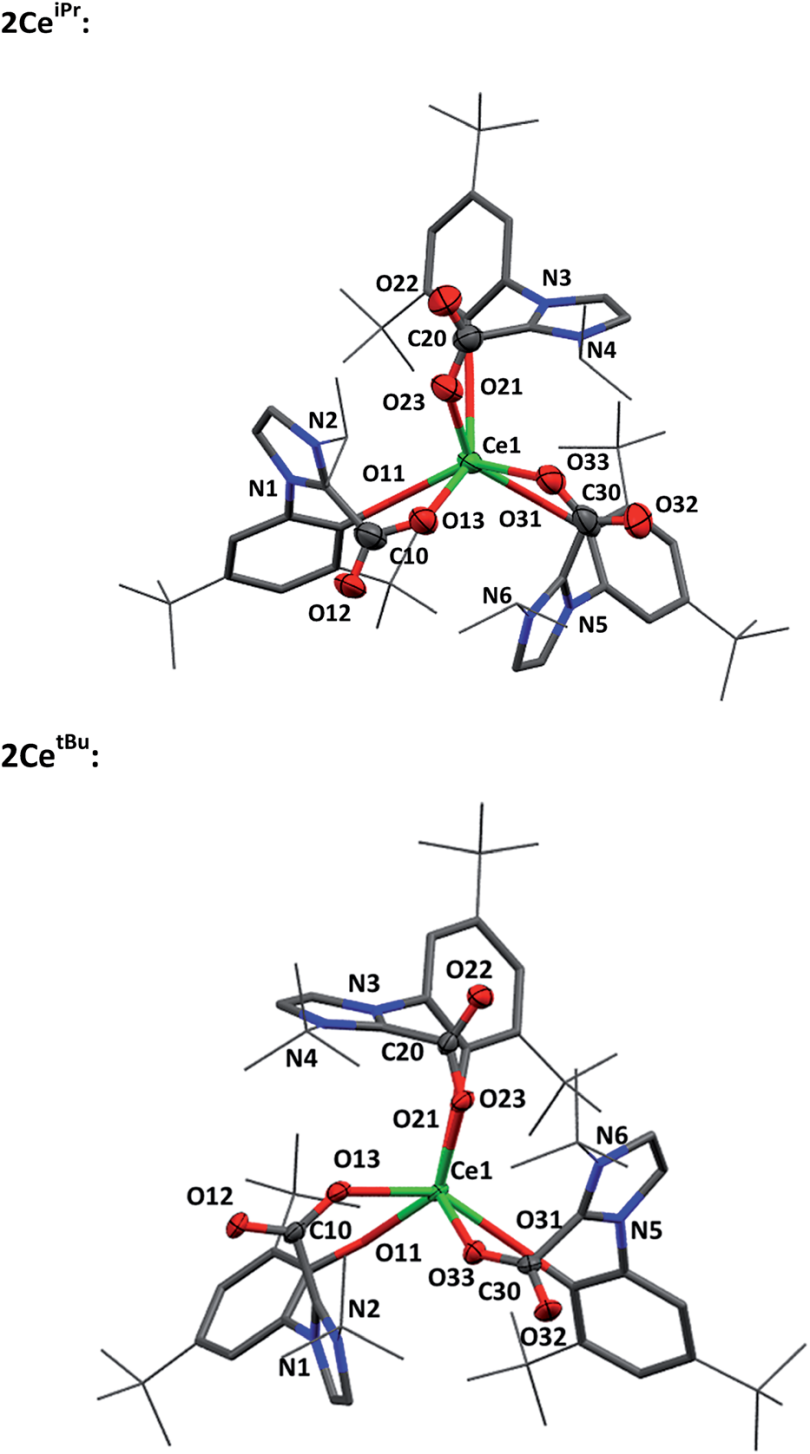
Molecular structures of **2Ce^iPr^** (upper) and **2Ce^tBu^** (lower) with carboxylate and Ce atoms shown at 50% ellipsoid probability, framework and peripheral carbon atoms drawn capped stick and wireframe respectively, and H and lattice solvent omitted for clarity. Selected distances (Å) and angles (°) for **2Ce^iPr^**: Ce1–O11 2.790(5), Ce1–O21 2.274(5), Ce1–O31 2.256(6), Ce1–O13 2.468(6), Ce1–O23 2.482(6), Ce1–O33 2.466(6), O11–Ce1–O21 97.18(19), O11–Ce1–O31 99.3(2), O21–Ce1–O31 94.8(2), O13–Ce1–O23 77.02(19), O13–Ce1–O33 76.68(19), O23–Ce1–O33 79.6(2), O11–Ce1–O13 75.72(19), O21–Ce1–O23 75.7(2), O31–Ce1–O33 75.9(2). For **2Ce^tBu^**: Ce1–O11 2.261(2), Ce1–O21 2.268(2), Ce1–O31 2.274(2), Ce1–O13 2.473(2), Ce1–O23 2.477(2), Ce1–O33 2.470(2), O11–Ce1–O21 95.38(8), O11–Ce1–O31 95.25(8), O21–Ce1–O31 97.20(8), O13–Ce1–O23 76.76(8), O13–Ce1–O33 80.89(8), O23–Ce1–O33 80.80(8), O11–Ce1–O13 75.50(8), O21–Ce1–O23 74.77(2), O31–Ce1–O33 75.03(8).

Single crystal X-ray analysis confirms that CO_2_ insertion products **2Ce^iPr^** and **2Ce^tBu^** have a pseudo-trigonal prismatic geometry with *C*_3_-symmetric *fac*-arrangement described by the average O^Ar^–Ce–O^Ar^ bond angles (97.03(10)° and 95.94(8)° respectively) and O^CO^–Ce–O^CO^ bond angles (77.77(10)° and 80.48(8)°) (Fig. 2). The average Ce–O^CO^ bond length is within the regular bond length range at (2.472(6) Å and 2.473(2) Å respectively) suggesting a strong degree of stabilisation despite an increase of metal chelate ring size from 6 to 8.

**Scheme 3:**
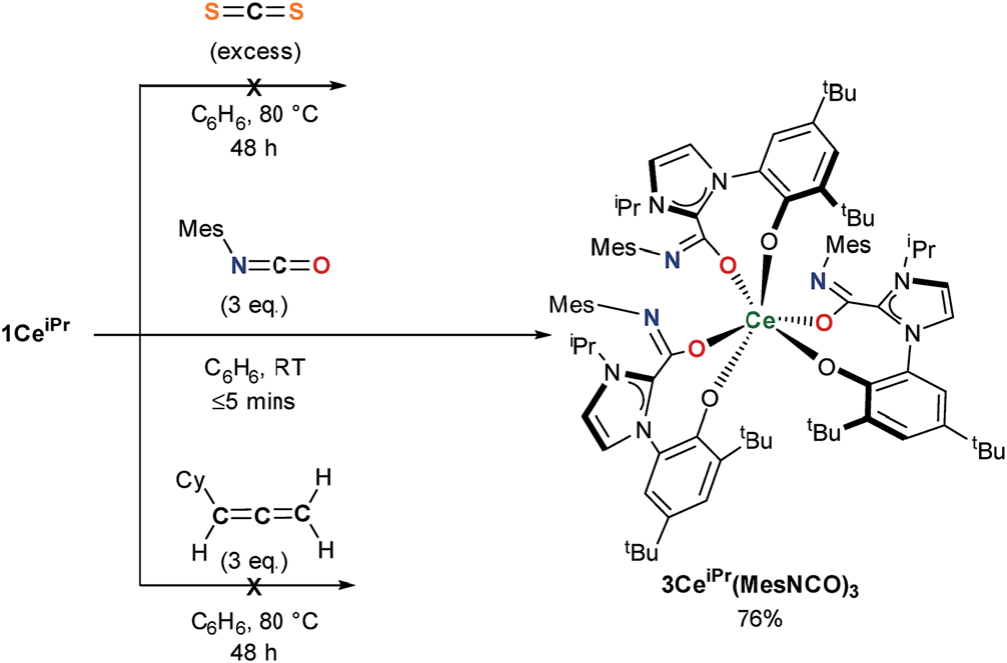
Treatment of **1Ce^iPr^** with reagents isoelectronic to CO_2_.

The substrate scope was further explored with carbon disulfide and other isoelectronic (hetero)allenes shown in Scheme 3. Interestingly, treatment of a benzene solution of **1Ce^iPr^** with excess carbon disulfide at temperatures up to 80 °C shows no reaction. This differs from the alkoxide-tethered carbene complex **D** for which the product arising from the insertion of CS_2_ into two (of the three) M–C bonds was characterized.^20^ The higher reactivity of CO_2_ compared to CS_2_ in this system is reasonable considering the stronger affinity of Ce for oxygen, and the lower dipole moment in the latter reagent.

Treatment of a benzene or THF solution of **1Ce^iPr^** with three equivalents of mesityl isocyanate (MesNCO) immediately results in the insertion of isocyanate into all three Ce–NHC bonds, affording a pale-yellow solution from which the tris-azoliumamidate **3Ce^iPr^(MesNCO)_3_** can be readily isolated as colourless microcrystalline powder in 76% yield, Scheme 3. No dimer or trimer isocyanate products were observed as a comparison to “free” NHC isocyanate chemistry.^9^ As could be expected, the non-polar and more sterically hindered cyclohexylallene shows no reactivity with **1Ce^iPr^**.

**Scheme 4:**
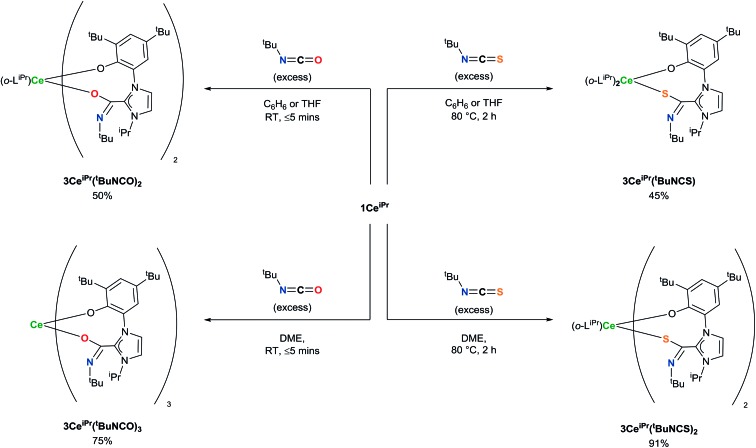
Differences in reactivity of **1Ce^iPr^** with sterically hindered isocyanates and isothiocyanates depending on solvent, and ligand substituents to afford **3Ce^iPr^(^t^BuNCO)_2_**, **3Ce^iPr^(*^t^*BuNCO)_3_**, **3Ce^iPr^(^t^BuNCS)**, and **3Ce^iPr^(^t^BuNCS)**.

In the reaction of **1Ce^iPr^** with three equivalents of *tert*-butyl isocyanate (^t^BuNCO) in benzene or THF, two molecules of isocyanate insert to form **3Ce^iPr^(^t^BuNCO)_2_**, however in DME solution, three molecules insert to form **3Ce^iPr^(^t^BuNCO)_3_** as a 3 : 1 mixture of the *fac*- and *mer*-isomers observable by ^1^H NMR spectroscopy, Scheme 4. We suggest that in the former two solvents, the steric bulk of the *tert*-butyl groups restricts access to the third equivalent, but the stronger, bidentate donor solvent DME increases the lability of the NHC groups, enabling three insertions to occur. If **1Ce^iPr^** is treated with 3 equivs of *tert*-butyl isothiocyanate (^t^BuNCS) at 80 °C in benzene or THF, a single equivalent of isothiocyanate inserts to form **3Ce^iPr^(^t^BuNCS)** while in DME two equivalents of isothiocyanate insert to form **3Ce^iPr^(^t^BuNCS)_2_**.

**Fig. 3:**
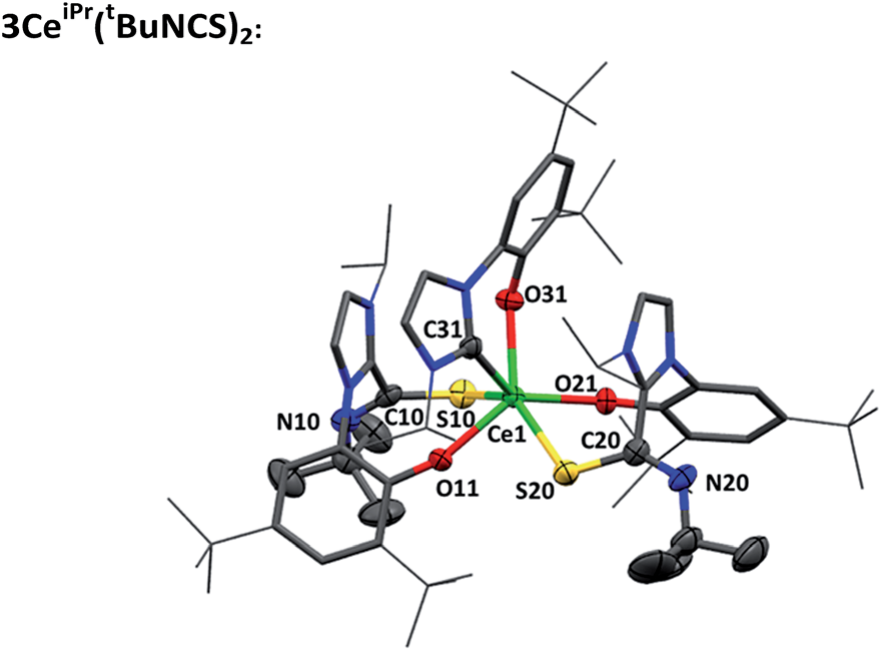
Molecular structure of **3Ce^iPr^(^t^BuNCS)_2_** with selected C and non-C/H atoms shown at 50% ellipsoid probability, framework and peripheral carbon atoms drawn capped stick and wireframe respectively, and H and lattice solvent omitted for clarity. Selected average distances (Å) and angles (°) for **3Ce^iPr^(^t^BuNCS)_2_**: Ce1–S10 2.996(12), Ce1–S20 3.048(12), Ce1–C31 2.716(4), Ce1–O11 2.280(3), Ce1–O21 2.273(3) Ce1–O31 2.277(3), S10–Ce1–S20 143.91(3), S10–Ce1–C31 126.96(9), S20–Ce1–C31 80.70(7), S10–Ce1–O11 78.71(8), S20–Ce1–O21 78.79(7), C31–Ce1–O31 69.93(11).

Single crystals of **3Ce^iPr^(^t^BuNCS)_2_** were grown by slow diffusion of heptane into a toluene solution. An X-ray diffraction study, Fig. 3, reveals a pseudo-trigonal prismatic molecular geometry at the metal center in the solid state. The Ce–S bond lengths average at 3.022 Å, and the Ce–C bond (2.716 Å) is only a little shorter than the average Ce–C bond length in the parent compound **1Ce^iPr^** (2.742 Å). The obtuse S–Ce–S bond angle (143.91°) and chelate angle of each bidentate ligand is within the expected range; S–Ce–O^Ar^ (78.75° avg.) and C–Ce–O^Ar^ (69.93°).

### Catalytic applications of **2Ce^R^** complexes

**Scheme 5:**
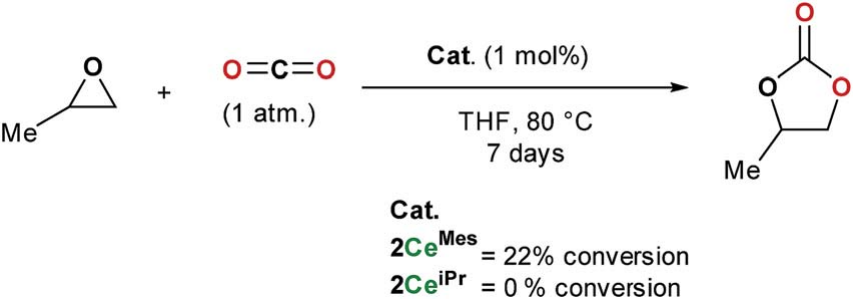
Catalytic formation of propylene carbonate from propylene oxide in an atmosphere of carbon dioxide using **2Ce^Mes^** and **2Ce^iPr^**.

The formation of cyclic carbonates from epoxides and carbon dioxide was chosen for a preliminary study of the catalytic activity of the tris(ligand) CO_2_ adducts **2Ce^iPr^** and **2Ce^Mes^**. Both free base NHCs and imidazolium carboxylates can be used as catalysts for the formation of cyclic carbonates from epoxides and carbon dioxide under high temperatures and pressures (up to 120 °C and 20 atm), while rare earth initiators are known to function at lower temperatures and/or pressures, a co-catalyst is usually required.^11^,^35^ Scheme 5 shows how under an atmosphere of carbon dioxide, 1 mol% of **2Ce^Mes^** catalyses the conversion of propylene oxide to propylene carbonate with 22% conversion at 80 °C in THF over 7 days, a much higher activity than the imidazolium carboxylates alone. On the other hand, the more compact **2Ce^iPr^** shows no reactivity. The solid-state structures show a higher steric congestion in the L^Mes^ adduct **1Ce^Mes^**, and IR and NMR spectroscopies confirm different ligand solution environments for **2Ce^Mes^**, suggesting both the Ce–C_carbene_ and Ce–C_CO_2__ interactions are weaker and more labile for the Mes system. We propose that the catalysis requires a combination of Lewis base type NHC–CO_2_ activation, and Lewis acid type Ce-epoxide activation.

### Synthesis of the heteroleptic substituted NHC analogues

**Scheme 6:**
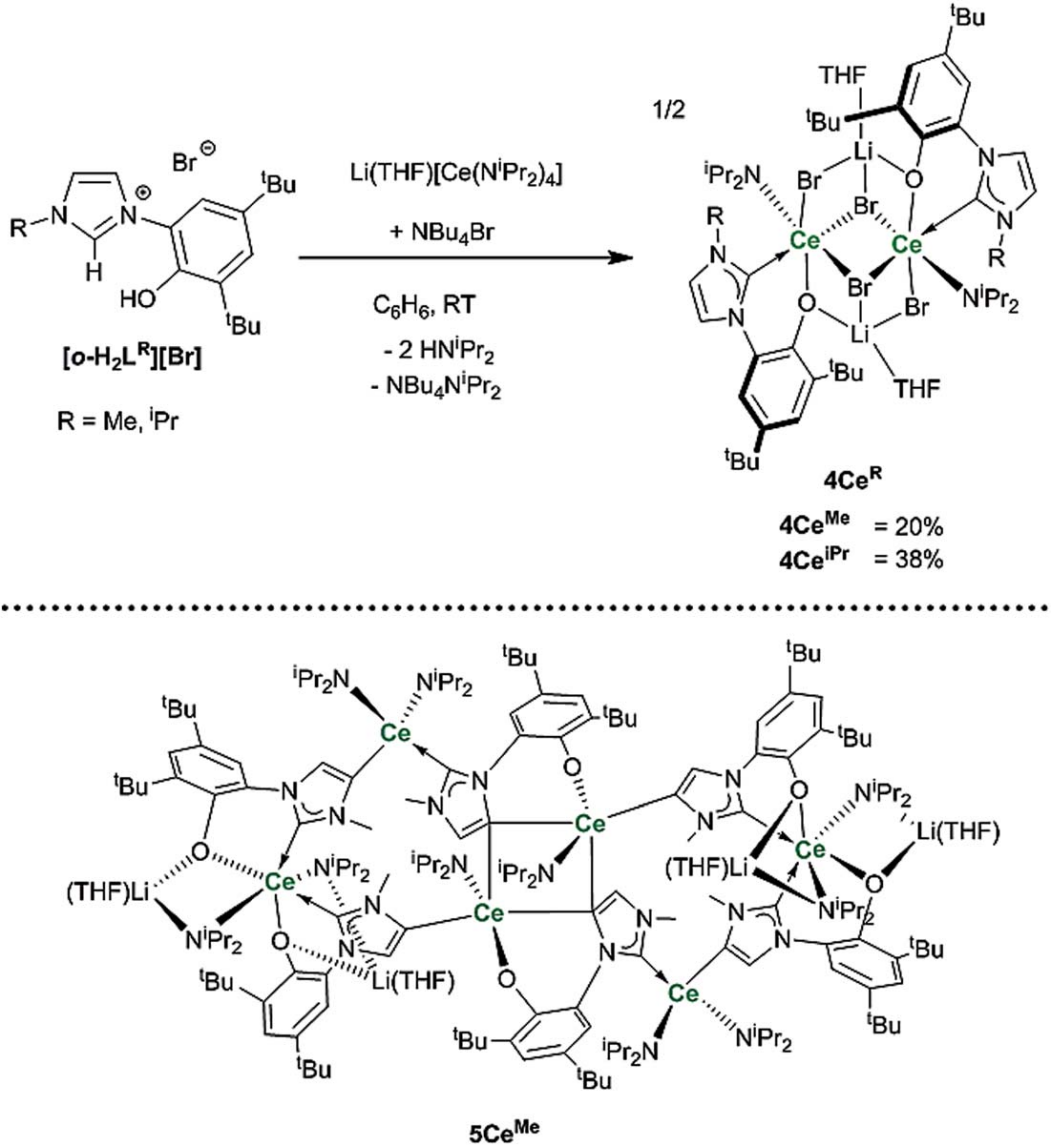
Reactions to target mono–NHC Ln complexes that afford **4Ce^Me^**, **4Ce^iPr^** and the hexanuclear **5Ce^Me^** that is the by-product isolated as single X-ray quality crystals for R = Me.

To target reactions with single equivalents of CO_2_, reactions designed to make complexes containing a single NHC ligand were carried out. The reactions of the ligands **[*o*-H_2_L^R^][Br]**, R = Me, ^i^Pr and equimolar amounts of Li(THF)[CeN(^i^Pr_2_)_4_] only afford clean material in low yields and significant decomposition can be observed. Adding an additional bromide source improves the yield of the mono-NHC–Ce complexes **4Ce^Me^** and **4Ce^iPr^** (Ce_2_Br_4_L^R^(^i^Pr_2_N)_2_Li_2_(THF)_2_) to a moderate level (20% and 38% respectively, see Scheme 6).

**Fig. 4:**
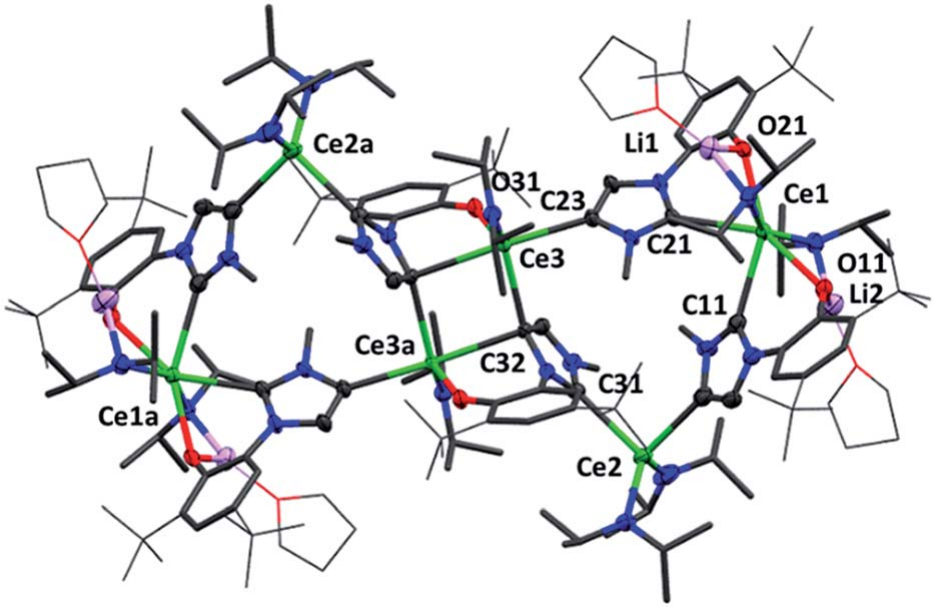
Molecular structure of **5Ce^Me^** with imidazolium, Ce, O and Li atoms shown at 50% ellipsoid probability, framework atoms drawn capped stick and coordinated solvents, peripheral carbon atoms wireframe, and H and lattice solvent omitted for clarity. Selected distances (Å) and angles (°) for **5Ce^Me^**: Ce1–C11 2.743(5), Ce1–C21 2.728(5), Ce2–C13 2.667(7), Ce2–Ce31 2.710(5), Ce3–C23 2.651(6), Ce3–C32 2.854(4), Ce3–C32a 2.685(6), Ce1–O11 2.517(4), Ce1–C21 2.462(4), Ce3–O31 2.232(3), C11–Ce1–O11 65.1(2), C11–Ce1–C21 82.1(2), C21–Ce1–O21 65.9(1), C13–Ce2–C31 94.4(2), Ce3–C32–Ce3a 98.5(1), C32–Ce3–C32a 81.5(1), C32–Ce3–O31 71.1(1), C32–Ce3–C23a 163.8(2).

Crystallographic analysis reveals a dimeric structure still containing unreacted base and lithium ions (see ESI†). A complicated bis(ligand) Li_4_Ce_6_ cluster **5Ce^Me^**, in which each ligand has been deprotonated at the NHC backbone (in the 4-position) yielding a dianionic OC ligand that bridges two cerium cations, is isolated in low yield as orange crystals that are suitable for single crystal diffraction studies (Fig. 4 and ESI†). Syntheses to target **4** or **5** in the absence of an additional bromide source, or from cerium bromide starting materials, yield only complicated mixtures of compounds in our hands.

### Synthesis of the *para*-aryloxide substituted NHC analogues

**Scheme 7:**
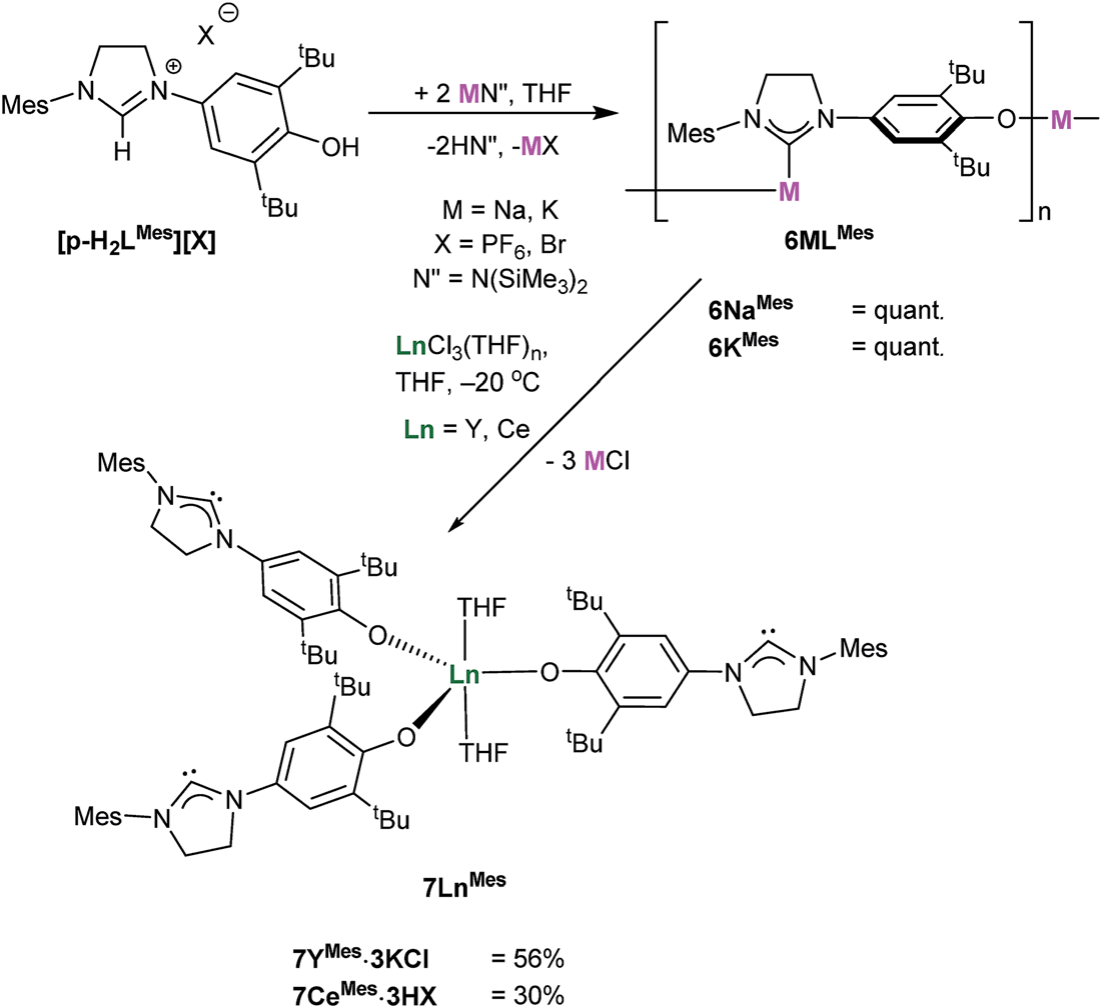
Reaction to target the *para*-ligand adducts **7Ln^Mes^** (Ln = Y, Ce).

The analogous complexes of the *para*-substituted aryloxide ligand *p*-L^R^ separate the Lewis acid and Lewis base centers, and thus offer a potential insight into the importance of the adjacent Ln centre and the nucleophilic NHC in the combined activation of CO_2_ and the other unsaturated substrates. A modification of Wang's proligand synthesis using saturated-backbone imidazoline analogues allows access to the *para*-functionalized proligand in 15% yield.^27^ Treatment of this *N*-mesityl functionalized proligand **[*p*-H_2_L^Mes^][X]**, (*p*-L^Mes^ = 4-O-2,6-^t^Bu_2_-C_6_H_2_(1-C{N(CH)_2_NMes}), X = PF_6_, Br) with either MN(SiMe_3_)_2_ (M = Na or K) in THF at room temperature affords the group 1 NHC salts **6M^Mes^** [(M(*p*-L^Mes^)]_*n*_, (M = Na, K) in quantitative yield, Scheme 7. The solid-state structures of both are polymeric, according to single crystal X-ray data, with **6Na^Mes^** displaying repeating C–[Na–(μ-ArO)_2_–Na]–C diamond units, while **6K^Mes^** displays a perpendicular ArO–K–C arrangement, see ESI.†

Salt **6M^Mes^** can be treated with YCl_3_ or CeCl_3_ at –20 °C to synthesise **7Ln^Mes^** (Ln(*p*-L^Mes^)_3_(THF)_2_ where Ln = Y, Ce) in 56% and 30% yield as yellow powders. Due to their high reactivity all the compounds start to degrade rapidly making further analysis difficult, and the complexes are best stored in their protonated form, *i.e.* [Ln(*p*-HL^Mes^)_3_(THF)_2_]X_3_.

Analysis of **7Y^Mes^** by ^13^C NMR spectroscopy reveals a characteristic carbene signal (*δ* 238.2 ppm) is observed as a singlet indicating that the carbene does not bind to yttrium in solution. These complexes were found to be extremely air sensitive, were only ever isolated as KCl and HCl salts and became highly insoluble in a range of solvents so were not pursued further (see ESI†).

## Conclusions

The tris(*ortho*-aryloxide–NHC) rare earth complexes Ln(L^R^)_3_ are readily isolated and are the thermodynamic sink in this system. Insertion of CO_2_ or a range of isoelectronic (hetero)allenes into the labile cerium carbene bond in Ce(L^R^)_3_ shows a dependence on solvent and N-R group on L^R^ that enables control of the degree of insertion. The CO_2_-insertion products form cleanly at ambient pressure, but only reversibly for the bulky mesityl substituted Ce(L^Mes^)_3_. The reversibility of the CO_2_ insertion appears to be crucial for further reactivity as only Ce(L^Mes^·CO_2_)_3_ is an active catalyst for the conversion of propylene oxide to propylene carbonate. Although yields in these preliminary tests using low temperatures and one atmosphere of CO_2_ are low, the catalyst is more active than a monodentate NHC, and when the ligands are better fit to the metal in Ce(L^iPr·CO_2_^)_3_, the complexes are inactive. We propose that the catalysis requires a combination of Lewis base type NHC–CO_2_ activation, and Lewis acid type Ce-epoxide activation.

Although the tris-ligand complexes are the thermodynamic sink in the system, the mono-ligand complexes can be isolated from reactions using LiCe(N^i^Pr_2_)_4_ as a starting material; LiBr adducts [Ce(L^R^)(N^i^Pr_2_)Br·LiBr(THF)]_2_ (R = Me, ^i^Pr) are reported, along with a hexanuclear N-heterocyclic dicarbene complex [Li_2_Ce_3_(OArC^Me^–H)_3_(N^i^Pr_2_)_5_(THF)_2_]_2_ which is formed as a by-product. The analogous *para*-aryloxide–NHC proligand *p*-L^Mes^ has been made for comparison. The group 1 salts [Na(*p*-L^Mes^)]_*n*_ and [K(*p*-L^Mes^)]_*n*_ form two different types of infinite coordination polymers through metal carbene-bonds. Synthesis of the analogous lanthanide *para*-aryloxide NHC complexes Ln(*p*-L^Mes^)_3_(THF)_2_ (Ln = Y, Ce) is possible but they are all highly reactive leading to rapid degradation. Therefore straightforward Lewis pair separated chemistry cannot usefully be carried out.

Further work is underway to use the *C*_3_-symmetric tris(*ortho*-aryloxide–NHC)–CO_2_ adducts in asymmetric catalysis and to expand the scope of the CO_2_ functionalisation.

## Experimental

### General details

All manipulations were carried out under a dry, oxygen-free atmosphere of nitrogen using standard Schlenk and glovebox technique. All gases were supplied by BOC gases UK. All glassware items, cannulae and Fisherbrand 1.2 μm retention glass microfibre filters were dried in a 170 °C oven overnight before use. Benzene and DME were distilled from potassium and stored over 4 Å molecular sieves. Hexane, heptane, THF, and toluene were degassed and purified by passage through activated 4 Å molecular sieves or activated alumina towers and stored over 4 Å molecular sieves. Deuterated solvents, benzene-*d*_6_, THF-*d*_8_ and pyridine-*d*_5_ were dried over potassium, vacuum-transferred, and freeze–pump–thaw degassed prior to use. ^1^H and ^13^C NMR spectra were recorded on Bruker AVA400, AVA500, or PRO500 spectrometers at 300 K. Chemical shifts are reported in parts per million, *δ*, referenced to residual proton resonances, and calibrated against external TMS. Infrared spectra were recorded on a Perkin Elmer Spectrum 65 FT-IR spectrometer as nujol mulls between KBr disks. Mass spectra were acquired using a SolariX FT-ICR (12 T) (Bruker UK Ltd) equipped with a Bruker APPI source. Samples were prepared as *ca.* 1 mM toluene solutions of the substrate. Elemental analyses were carried out at London Metropolitan University, London, UK.

NaN(SiMe_3_)_2_, KN(SiMe_3_)_2_,^36^ and the **[*o*-H_2_L^R^][Br]**^27^,^28^ pro-ligands were prepared according to the literature procedures. YCl_3_(H_2_O)_*n*_ and LnCl_3_(H_2_O)_*n*_ were purchased and stirred overnight with TMSCl (40 equiv.) in THF before vacuum drying for several hours.

### General procedure 1 – synthesis of **1Ln^R^**

To a suspension of **[*o*-H_2_L^R^][Br]** (3 equiv.) in DME (0.1 M) KN(SiMe_3_)_2_ (6 equiv.) was added and the resulting mixture was stirred for 5 min at –20 °C while and warmed to room temperature. LnCl_3_(THF)_*n*_ (1 eq.) was added, and the resulting mixture was stirred at room temperature for 2 h. Volatiles were removed under reduced pressure, the crude product was extracted three times with hexane and the combined filtrates were concentrated to saturation and cooled to –20 °C overnight. The resulting suspension was filtered and the solid collected and dried under vacuum to give the title compound which was stored at –20 °C under a nitrogen atmosphere.

#### **1Ce^iPr^**

Using general procedure 1 – 3-(3,5-di-*tert*-butyl-2-hydroxyphenyl)-1-isopropyl-1*H*-imidazol-3-ium bromide **[*o*-H_2_L^iPr^][Br]** (11.82 g, 30 mmol), KN(SiMe_3_)_2_ (11.97 g, 60 mmol), CeCl_3_(THF)_1.15_ (3.29 g, 10 mmol) and DME (100 mL) gave after recrystallization the title compound **1Ce^iPr^** as a yellow solid (8.17 g, 7.6 mmol, 76%). X-ray quality crystals were grown from a concentrated hexane solution at –20 °C over 1 week. ^1^H NMR (500 MHz, C_6_D_6_) *δ*_H_: –9.76 (3H, s, CH(C*H*_3_)), –6.89 (3H, s, CH(C*H*_3_)), –4.37 (3H, s, CH(C*H*_3_)), –3.38 (9H, s, C(C*H*_3_)_3_), –1.62 (3H, s, CH(C*H*_3_)), 0.47 (3H, s, CH(C*H*_3_)), 0.77 (s, 1H, C*H*), 1.51 (9H, s, C(C*H*_3_)_3_), 1.57 (9H, s, C(C*H*_3_)_3_), 1.75 (1H, s, C*H*), 1.76 (1H, s, C*H*), 2.10 (9H, s, C(C*H*_3_)_3_), 2.31 (9H, s, C(C*H*_3_)_3_), 3.37–3.32 (9H, m, C(C*H*_3_)_3_), 3.43 (3H, s, CH(C*H*_3_)), 5.97 (1H, s, C*H*), 6.70 (1H, s, C*H*), 7.08 (1H, app d, *J* 2.7, C*H*), 7.08 (1H, app d, *J* 2.7, C*H*), 7.62 (1H, s, C*H*), 7.70 (1H, app d, *J* 2.7, C*H*), 8.91 (1H, s, C*H*), 9.75 (1H, s, C*H*), 10.18 (2H, m, 2 × C*H*), 10.39 (1H, s, C*H*), 11.01 (1H, s, C*H*), 11.22 (1H, s, C*H*). ^13^C{^1^H} NMR (126 MHz, C_6_D_6_) *δ*_C_: 14.4, 21.7, 22.3, 23.1, 24.6, 24.7, 30.1, 30.1, 31.9, 32.0, 32.5, 33.4, 33.9, 34.2, 35.4, 36.2, 36.5, 39.4, 41.0, 41.8, 46.4, 51.4, 114.4, 115.6, 118.9, 119.3, 119.5, 121.3, 122.2, 122.6, 122.7, 123.8, 124.2, 129.7, 131.4, 138.8, 140.3, 140.5, 141.9, 146.2, 147.4, 147.7, 148.6, 154.0, 155.8, 162.9, 174.8, 187.8, 192.2. Elemental analysis C_60_H_87_CeO_3_N_6_: C 66.70%, H 8.12%, N 7.78% calculated. C 66.72%, H 8.13%, N 7.78% found; APPI^+^ C_60_H_87_CeN_6_O_3_^+^ [M]^+^ requires 1079.5894, found 1079.5711 (–17.0 ppm).

#### **1Ce^tBu^**

Using general procedure 1 – 1-(*tert*-butyl)-3-(3,5-di-*tert*-butyl-2-hydroxyphenyl)-1*H*-imidazol-3-ium bromide **[*o*-H_2_L^tBu^][Br]** (306 mg, 0.75 mmol), KN(SiMe_3_)_2_ (300 mg, 1.5 mmol), CeCl_3_(THF)_1.15_ (80 mg, 0.25 mmol) and DME (2.5 mL) gave after recrystallization title compound **1Ce^tBu^** as a yellow solid (70 mg, 0.0625 mmol, 25%). ^1^H NMR (400 MHz, C_6_D_6_) *δ*_H_: –18.43 (9H, s, C(C*H*_3_)_3_), –9.47 (9H, s, C(C*H*_3_)_3_), –4.21 (9H, s, C(C*H*_3_)_3_), –3.27 (9H, s, C(C*H*_3_)_3_), –1.25 (9H, s, C(C*H*_3_)_3_), 1.18 (9H, s, C(C*H*_3_)_3_), 2.41 (9H, s, C(C*H*_3_)_3_), 4.02 (9H, s, C(C*H*_3_)_3_), 4.88 (1H, s, Ar*H*), 6.23 (1H, s, Ar*H*), 7.45 (1H, s, Ar*H*), 8.66 (1H, s, Ar*H*), 9.30–9.38 (10H, m, Ar*H* + C(C*H*_3_)_3_), 9.63 (1H, s), 9.68 (1H, s), 11.97 (1H, s), 16.43 (1H, s), 16.84 (1H, s). ^13^C{^1^H} NMR (126 MHz, C_6_D_6_) *δ*_C_: 23.9, 25.3, 27.0, 29.0, 29.1, 30.1, 33.8, 34.2, 35.5, 35.7, 36.7, 36.9, 37.9, 38.9, 48.2, 51.8, 53.0, 57.4, 113.5, 115.1, 117.1, 118.1, 118.8, 120.6, 121.4, 123.0, 123.7, 124.3, 125.5, 130.3, 132.5, 133.4, 134.5, 137.9, 140.6, 142.4, 144.7, 147.0, 149.9, 157.3, 160.8, 163.1, 171.1, 206.2, 213.0. APPI^+^ C_63_H_93_CeN_6_O_3_^+^ [M]^+^ requires 1121.6364, found 1121.6333 (–2.7 ppm). After several attempts, this compound did not give satisfactory elemental analysis results, presumably because of its thermal sensitivity.

#### **1Ce^Mes^**

Using general procedure 1 – 3-(3,5-di-*tert*-butyl-2-hydroxyphenyl)-1-mesityl-1*H*-imidazol-3-ium **[*o*-H_2_L^Mes^][Br]** (353 mg, 0.75 mmol), KN(SiMe_3_)_2_ (300 mg, 1.5 mmol), CeCl_3_(THF)_1.15_ (80 mg, 0.25 mmol) and DME (2.5 mL) gave after extraction and recrystallization in benzene title compound **1Ce^Mes^** as a yellow solid (42 mg, 0.037 mmol, 15%). X-ray quality crystals were grown from a concentrated benzene solution over 1 week at room temperature. ^1^H NMR (400 MHz, C_6_D_6_) *δ*_H_: –8.50 (9H, ArC*H*_3_), –3.80 (27H, s, C(C*H*_3_)_3_), 1.51 (9H, s, ArC*H*_3_), 2.13 (3H, s, Ar*H*), 2.80 (27H, s, C(C*H*_3_)_3_), 7.92 (9H, s, ArC*H*_3_), 8.33 (3H, s, Ar*H*), 8.53 (3H, s, Ar*H*), 9.07 (3H, s, Ar*H*), 11.54 (3H, s, Ar*H*), 12.01 (3H, s, Ar*H*). ^13^C{^1^H} NMR (126 MHz, C_6_D_6_) *δ*_C_: 20.1 (Ar*C*H_3_), 21.8 (Ar*C*H_3_), 25.4 (ArC(*C*H_3_)_3_), 32.7 (Ar*C*H_3_), 34.9 (ArC(*C*H_3_)_3_), 36.0 (ArC(*C*H_3_)_3_), 122.4 (Ar*C*), 123.2 (Im*C*), 123.8 (Ar*C*), 124.8 (Ar*C*), 125.4 (Ar*C*), 129.5 (Ar*C*), 130.5 (Ar*C*), 130.5 (Ar*C*), 133.0 (Im*C*), 135.1 (Ar*C*), 135.7 (Ar*C*), 138.3 (Ar*C*), 139.8 (Ar*C*), 147.9 (Ar*C*), 148.3 (Ar*C*), 184.2 (N*C*N). Elemental analysis C_78_H_88_CeO_3_N_6_: C 71.58%, H 7.62%, N 6.42% calculated. C 71.43%, H 7.76%, N 6.31% found; APPI^+^ C_78_H_99_CeN_6_O_3_^+^ [M]^+^ requires 1307.6833, found 1307.6810 (–1.7 ppm).

#### **1Sm^iPr^**

Using general procedure 1 – 3-(3,5-di-*tert*-butyl-2-hydroxyphenyl)-1-isopropyl-1*H*-imidazol-3-ium bromide **[*o*-H_2_L^iPr^][Br]** (296 mg, 0.75 mmol), KN(SiMe_3_)_2_ (300 mg, 1.5 mmol), SmCl_3_(THF)_2_ (100 mg, 0.1575 mmol) and DME (2.5 mL) gave after recrystallization title compound **1Sm^iPr^** as a yellow solid (171 mg, 7.6 mmol, 63%). ^1^H NMR (400 MHz, C_6_D_6_) *δ*_H_: –9.17–(–9.07) (1H, m, C*H*(CH_3_)), –4.24 (3H, app d, *J* 5.8, CH(C*H*_3_)), –2.42 (3H, app d, *J* 5.5, CH(C*H*_3_)), –1.13 (9H, s, C(C*H*_3_)), –0.85–(–0.73) (6H, m, 2*x*CH(C*H*_3_)), –0.63–(–0.59) (1H, m, C*H*(CH_3_)), 1.08 (9H, s, C(C*H*_3_)), 1.65–1.70 (3H, m, CH(C*H*_3_)), 1.81 (10H, s, C(C*H*_3_)), 1.85 (10H, s, C(C*H*_3_)), 2.02 (10H, s, C(C*H*_3_)), 2.39 (10H, s, C(C*H*_3_)), 3.24 (3H, app d, *J* 5.5, CH(C*H*_3_)), 4.99 (1H, app p, *J* 6.8, C*H*(CH_3_)), 5.54 (1H, app d, *J* 1.8, Ar*H*), 6.05 (1H, app d, *J* 1.7, Ar*H*), 7.26 (1H, app d, *J* 1.7, Ar*H*), 7.90 (1H, app d, *J* 2.5, Im*H*), 8.06 (1H, app d, *J* 1.7, Ar*H*), 8.14 (1H, app d, *J* 2.4, Im*H*), 8.15 (1H, app d, *J* 2.4, Im*H*), 8.32 (1H, app d, *J* 2.6, Im*H*), 8.43 (1H, app d, *J* 1.8, Ar*H*), 8.46 (1H, app d *J* 2.6, Im*H*), 8.53 (1H, app d, *J* 1.7, Ar*H*), 8.94 (1H, app d, *J* 2.5, Im*H*). Elemental analysis C_60_H_87_SmO_3_N_6_: C 66.07%, H 8.04%, N 7.70% calculated. C 60.10%, H 8.33%, N 7.54% found. APPI^+^ C_60_H_87_SmN_6_O_3_^+^ [M]^+^ requires 1091.6037, found 1091.6076 (+3.6 ppm).

#### **1Eu^iPr^**

Using general procedure 1 – 3-(3,5-di-*tert*-butyl-2-hydroxyphenyl)-1-isopropyl-1*H*-imidazol-3-ium bromide **[*o*-H_2_L^iPr^][Br]** (296 mg, 0.75 mmol), KN(SiMe_3_)_2_ (300 mg, 1.5 mmol), EuCl_3_(THF)_2.5_ (110 mg, 0.1575 mmol) and DME (2.5 mL) gave after recrystallization title compound **1Eu^iPr^** as an orange-red solid (121 mg, 0.11 mmol, 45%). ^1^H NMR (400 MHz, C_6_D_6_) *δ*_H_: –21.12 (1H, s), –14.06 (9H, s, C(C*H*_3_)_3_), –11.59 (3H, s, CH(C*H*_3_)), –6.76 (9H, s, C(C*H*_3_)_3_), –5.94 (1H, s, C*H*), –5.90 (1H, s, C*H*), –5.38 (1H, s, C*H*), –2.60 (1H, s, C*H*), –1.63 (9H, s, C(C*H*_3_)_3_), –1.48 (9H, s, C(C*H*_3_)_3_), –1.44 (9H, s, C(C*H*_3_)_3_), –0.72 (1H, s, C*H*), –0.64 (1H, s, C*H*), –1.77 (3H, s, CH(C*H*_3_)), 3.26 (1H, s, C*H*), 4.76 (3H, s, CH(C*H*_3_)), 6.07 (1H, s, C*H*), 6.20 (1H, s, C*H*), 7.20 (1H, s, C*H*), 7.39 (1H, s, C*H*), 11.89 (9H, s, C(C*H*_3_)_3_), 15.05 (3H, s, CH(C*H*_3_)), 15.85 (1H, s, C*H*), 17.88 (1H, s, C*H*), 24.72 (3H, s, CH(C*H*_3_)), 33.23 (3H, s, CH(C*H*_3_)), 49.20 (1H, s, C*H*), 96.66 (1H, s, C*H*). Elemental analysis C_60_H_87_EuO_3_N_6_: C 65.97%, H 8.03%, N 7.69% calculated. C 66.00%, H 8.01%, N 7.67% found; APPI^+^ C_60_H_87_EuN_6_O_3_^+^ [M]^+^ requires 1092.6052, found 1092.6095 (+3.9 ppm).

### General procedure 2 – synthesis of **2Ce^R^**

A solution of **1Ce^R^** (3 equiv.) in benzene, toluene, hexane or THF (0.5 M) was freeze–pump–thaw degassed 3 times and exposed to an atmosphere of dry CO_2_ in a Teflon-valved ampoule. The solvent was removed under reduced pressure, and the crude product was extracted with toluene and concentrated to saturation and cooled to –30 °C overnight. The resulting suspension was filtered and dried under vacuum to yield the title compound which was stored at –20 °C under a nitrogen atmosphere.

#### **2Ce^iPr^**

Using general procedure 2 – **1Ce^iPr^** (3.0 g, 2.78 mmol) in toluene (50 mL) was charged with an atmosphere of CO_2_ and after recrystallization gave the title product **2Ce^iPr^** as a colourless solid (2.05 g, 1.69 mmol, 61%). Colourless crystals suitable for X-ray diffraction were grown from slow diffusion of hexanes into a concentrated THF solution. ^1^H NMR (400 MHz, C_6_D_6_) *δ*_H_: 0.91 (27H, s, C(C*H*_3_)_3_), 1.27–1.31 (9H, m, CH(C*H*_3_)_a_(CH_3_)_b_), 1.72–1.76 (9H, m, (CH(C*H*_3_)_a_(CH_3_)_b_), 2.52 (27H, s, C(C*H*_3_)_3_), 4.46–4.50 (3H, m, Ar*H*), 4.62–4.66 (3H, m, Ar*H*), 5.97 (3H, d, *J* 2.6, Im*H*), 7.59 (3H, d, *J* 2.6, Im*H*), 8.42 (3H, m, C*H*(CH_3_)_a_(CH_3_)_b_). ^13^C{^1^H} NMR (126 MHz, C_6_D_6_) *δ*_H_: 21.4 (CH(*C*H_3_)_a_(CH_3_)_b_), 24.6 (CH(CH_3_)_a_(*C*H_3_)_b_), 30.1 (C(*C*H_3_)_3_), 31.5 (C(*C*H_3_)_3_), 33.6 (*C*(CH_3_)_3_), 36.7 (*C*(CH_3_)_3_), 53.1 (*C*H(CH_3_)_a_(CH_3_)_b_), 112.4 (N*C*N), 119.1 (Ar*C*), 119.5 (Ar*C*), 123.6 (Im*C*), 125.2 (Im*C*), 134.6 (Ar*C*), 139.4 (Ar*C*), 143.2 (Ar*C*), 154.9 (Ar*C*), 173.1 (O*C*O). *ν*_max_ (nujol mull): 1666. Elemental analysis C_63_H_87_CeO_9_N_6_: C 62.25%, H 7.46%, N 6.91% calculated. C 62.36%, H 7.58%, N 7.08% found.

#### **2Ce^tBu^**

Using general procedure 2 – **1Ce^tBu^** (32 mg, 0.022 mmol) and THF (1 mL) was charged with an atmosphere of CO_2_ and after recrystallization gave the title product **2Ce^tBu^** as a colourless solid (20 mg, 16 mmol, 72%). ^1^H NMR (400 MHz, C_6_D_6_) *δ*_H_: 0.87 (27H, s, C(C*H*_3_)_3_), 2.60 (27H, s, C(C*H*_3_)_3_), 2.94 (27H, s, C(C*H*_3_)_3_), 4.58–4.62 (3H, m, Ar*H*), 4.84–4.88 (3H, m, Ar*H*), 5.91 (3H, d, *J* 2.5, Im*H*), 7.69 (3H, d, *J* 2.5, Im*H*). ^13^C{^1^H} NMR (126 MHz, C_6_D_6_) *δ*_C_: 30.0 (C(*C*H_3_)_3_), 30.1 (C(*C*H_3_)_3_), 31.1 (C(*C*H_3_)_3_), 33.2 (*C*(CH_3_)_3_), 36.4 (*C*(CH_3_)_3_), 62.5 (N*C*(CH_3_)_3_), 113.9 (Ar*C*), 118.7 (Ar*C*), 120.4 (Ar*C*), 122.9 (Im*C*), 125.5 (Im*C*), 133.9 (Ar*C*), 141.8 (Ar*C*), 143.6 (Ar*C*), 160.8 (Ar*C*(2)OCe), 173.5 (O*C*O). Elemental analysis C_66_H_93_CeO_9_N_6_: C 62.25%, H 7.46%, N 6.91% calculated. C 62.36%, H 7.58%, N 7.08% found.

#### **2Ce^Mes^**

Using a modification of general procedure 2 – **1Ce^Mes^** (25 mg, 0.022 mmol) and THF (1 mL) was charged with an atmosphere of CO_2_ and the resulting solution was left to slow evaporate to give the title product **2Ce^Mes^** as a bright yellow solid (31 mg, 0.022 mmol, 99%). ^1^H NMR (400 MHz, *d*_8_-THF) *δ*_H_: –9.96 (3H, br. s, CH_3_), –6.09 (9H, br. s, C(C*H*_3_)_3_), –5.17 (9H, br. S, C(C*H*_3_)_3_), –2.95 (9H, br. s, C(C*H*_3_)_3_), –2.11 (3H, br. s, C*H*_3_), 0.82 (9H, br. s, C(C*H*_3_)_3_), 0.97 (3H, br. s, C*H*_3_), 1.13 (9H, br. s, C(C*H*_3_)_3_), 2.64 (12H, app. br. s, C(C*H*_3_)_3_ + C*H*_3_), 3.05 (1H, br. s, C*H*), 3.51 (1H, br. s, C*H*), 4.46 (1H, br. s, C*H*), 4.74 (6H, app. br. s, 2 × C*H*_3_), 5.61 (1H, br. s, C*H*), 5.72 (1H, br. s, C*H*), 6.06 (1H, br. s, C*H*), 6.28 (3H, br. s, C*H*_3_), 7.04 (1H, br. s, C*H*), 7.51 (1H, br. s, C*H*), 8.36 (1H, br. s, C*H*), 8.61 (1H, br. s, C*H*), 9.10 (3H, br. s), 10.61 (1H, br. s, C*H*), 12.01 (1H, br. s, C*H*), 12.23 (1H, br. s, C*H*), 12.59 (1H, br. s, C*H*), 13.00–3.60 (4H, m, C*H*_3_ + C*H*). Three C*H* resonances could not be located. ^13^C{^1^H} NMR (126 MHz, *d*_8_-THF) *δ*_C_: 7.3, 13.3, 16.7, 17.4, 19.5, 19.7, 20.0, 20.3, 21.1, 21.5, 22.8, 23.4, 28.2, 29.9, 30.6, 32.0, 32.5, 33.8, 34.2, 34.5, 36.3, 36.5, 113.9, 119.3, 119.5, 120.6, 121.0, 121.7, 122.4, 123.1, 123.2, 124.8, 126.3, 126.6, 127.4, 127.7, 128.1, 129.5, 130.1, 130.9, 131.8, 132.2, 132.6, 133.2, 134.3, 134.7, 135.3, 135.7, 135.9, 136.7, 137.5, 138.5, 139.3, 140.1, 141.0, 141.0, 141.5, 141.8, 142.4, 143.4, 145.7, 148.0, 160.0, 163.3, 164.7, 169.6, 170.9, 175.4, 180.4, 200.2. *ν*_max_ (nujol mull): 1678, 1716. Elemental analysis C_81_H_99_CeO_9_N_6_: C 67.52% H 6.93% N 5.83% calculated. C 67.21% H 7.25% N 5.66% found.

#### **3Ce^iPr^(MesNCO)_3_**

To a solution of **1Ce^iPr^** (108 mg, 0.1 mmol) in C_6_H_6_ (2 mL), MesNCO (48 mg, 0.03 mmol) was added and stirred for 15 min. The reaction mixture was filtered and cooled to –30 °C and the title product was isolated as a colourless powder by filtration of the solvents and drying under vacuum (123 mg, 79%). ^1^H NMR (500 MHz, C_6_D_6_) *δ*_H_: –6.53 (9H, s, C(C*H*_3_)_3_), –5.17 (3H, s, C*H*_3_), –4.52 (3H, s, C*H*_3_), –3.89 (3H, s, C*H*_3_), –0.21 (3H, s, C*H*_3_), 1.05 (3H, s, C*H*_3_), 1.10 (3H, s, C*H*_3_), 1.37 (9H, s, C(C*H*_3_)_3_), 1.53 (6H, s, Mes(2,6)C*H*_3_), 1.72 (2H, s, Mes(3,5)*H*), 1.99 (9H, s, C(C*H*_3_)_3_), 2.00 (6H, s, Mes(2,6)C*H*_3_), 2.02 (6H, s, Mes(2,6)C*H*_3_), 2.22 (3H, s, C*H*_3_), 2.56 (9H, s, C(C*H*_3_)_3_), 2.58 (3H, s, C*H*_3_), 2.64 (9H, s, C(C*H*_3_)_3_), 2.99 (1H, s, C*H*), 3.26 (1H, s, C*H*), 3.44 (1H, s, C*H*), 3.64 (2H, s, Mes(3,5)*H*), 4.34 (1H, s, C*H*), 4.87 (1H, s, C*H*), 5.86 (1H, s, C*H*), 6.52 (2H, s, Mes(3,5)*H*), 6.62 (1H, s, C*H*), 6.79 (3H, s, C*H*_3_), 7.27 (1H, s, C*H*_3_), 7.35 (1H, s, C*H*), 7.71 (1H, s, C*H*), 9.07 (1H, s, C*H*), 10.04 (1H, s, C*H*), 10.58 (9H, s, C(C*H*_3_)_3_), 10.64 (1H, s, C*H*), 10.85 (1H, s, C*H*), 12.62 (1H, s, C*H*). Elemental analysis C_90_H_120_CeN_9_O_6_: C 69.11%, H 7.73%, N 8.06% calculated. C 69.09%, H 8.11%, N 7.93% found; APPI^+^ C_90_H_121_CeN_9_O_6_^+^ [M + H]^+^ requires 1563.8494, found 1563.8419 (–4.8 ppm).

#### **3Ce^iPr^(^t^BuNCO)_3_**

To a solution of **1Ce^iPr^** (108 mg, 0.1 mmol) in DME (2 mL), ^t^BuNCO (20 mg, 0.3 mmol) was added and stirred for 15 min. The reaction mixture was filtered into hexane (1 mL) and cooled to –30 °C and the title product was isolated as a colourless powder by filtration of the solvents and drying under vacuum (126 mg, 91%). ^1^H NMR (500 MHz, C_6_D_6_) **(*fac*)-3Ce^iPr^(^t^BuNCO)_3_***δ*_H_: –6.17 (27H, s), –5.18 (9H, s), –3.72 (27H, s), –3.48 (3H, s), 0.50 (9H, s), 5.52 (27H, s), 9.91 (3H, s), 12.68 (3H, s), 17.99 (3H, s), 21.06 (3H, s). **(*mer*)-3Ce^iPr^(^t^BuNCO)_3_***δ*_H_: –12.69 (1H, s), –12.11 (9H, s), –7.69 (3H, s), –6.97 (3H, s), –5.96 (9H, s), –5.39 (9H, s), –5.00 (3H, s), –4.86 (3H, s), –2.67 (1H, s), –2.26 (9H, s), –2.08 (1H, s), –1.45 (1H, s), –1.09 (9H, s), –0.95 (3H, s), 0.14 (9H, s), 1.82 (3H, s), 2.11 (1H, s), 5.11 (9H, s), 5.26 (9H, s), 5.36 (9H, s), 5.94 (1H, s), 8.73 (1H, s), 9.24 (3H, s), 9.91 (1H, s), 12.01 (1H, s), 12.25 (1H, s), 12.62 (1H, s), 16.82 (1H, s), 17.31 (1H, s), 19.71 (1H, s), 20.30 (1H, s). APPI^+^ C_75_H_116_CeN_9_O_7_^+^ [M + H_2_O]^+^ requires 1394.8052, found 1394.8426 (+26.8 ppm). Elemental analysis C_77_H_114_CeN_9_O_6_: C 65.38%, H 8.34%, N 9.15% calculated. C 65.52%, H 8.45%, N 8.98% found.

#### **3Ce^iPr^(^t^BuNCO)_2_**

To a solution of **1Ce^iPr^** (108 mg, 0.1 mmol) in C_6_H_6_ or THF (2 mL), ^t^BuNCO (20 mg, 0.3 mmol) was added and stirred for 15 min. The reaction mixture was filtered into hexane (1 mL) and cooled to –30 °C and the title product was isolated as a pale-yellow powder by filtration of the solvents and drying under vacuum (76 mg, 59%). ^1^H NMR (500 MHz, C_6_D_6_) *δ*_H_: –6.95 (9H, s), –4.75 (3H, s), –2.95 (1H, s), –2.62 (3H, s), –1.00 (9H, s), –0.1, (9H, s), 0.13 (3H, s), 0.19 (9H, s), 0.59–0.64 (3H, m), 0.66 (1H, s), 1.05 (1H, s), 1.17 (3H, s), 1.35 (1H, s), 1.44 (1H, s), 1.77 (9H, s), 2.31 (9H, s), 2.52 (9H, s), 3.42 (1H, s), 4.15 (1H, s), 5.96 (3H, s), 6.28 (1H, s), 7.03 (1H, s), 7.24 (1H, s), 9.06 (1H, s), 9.38 (9H, s), 9.88 (1H, s), 10.38 (1H, s), 10.50 (1H, s), 12.30 (1H, s). APPI^+^ C_70_H_106_CeN_8_O_5_^+^ [M + H]^+^ requires 1278.7341, found 1278.7213 (–10.0 ppm). Elemental analysis C_70_H_105_CeN_8_O_5_: C 65.75%, H 8.28%, N 8.75% calculated. C 65.50%, H 8.58%, N 8.64% found.

#### **3Ce^iPr^(^t^BuNCS)_2_**

To a solution of **1Ce^iPr^** (108 mg, 0.1 mmol) in DME (2 mL), ^*t*^BuNCS (34 μL, 0.3 mmol) was added and stirred for 2 h at 80 °C. The reaction mixture was cooled to room temperature then evaporated to dryness. Colourless X-ray quality crystals were grown by diffusion of heptane into a toluene solution of the crude product, and isolated by decanting (89 mg, 68%). ^1^H NMR (500 MHz, C_6_D_6_) *δ*_H_: –9.13 (1H, s), –8.58 (3H, s), –8.29 (1H, s), –6.14 (3H, s), –4.01 (3H, s), –3.97 (9H, s), –3.53 (3H, s), –3.00 (9H, s), –2.28 (9H, s), –1.34 (1H, s), 0.23 (9H, s), 1.38 (1H, s), 1.68 (9H, s), 3.13 (1H, s), 3.24 (3H, s), 3.25 (9H, s), 4.78 (1H, s), 6.07 (1H, s), 8.29 (1H, s), 8.37 (1H, s), 9.15 (9H, s), 9.76 (1H, s), 9.90 (1H, s), 10.86 (1H, s), 13.99 (1H, s), 14.23 (3H, s), 17.32 (1H, s), 18.17 (9H, s), 52.70 (1H br. s). Elemental analysis C_70_H_105_CeN_8_O_3_S_2_: C 64.14%, H 8.07%, N 8.55% calculated. C 64.17%, H 8.35%, N 8.24% found. APPI^+^ C_70_H_106_CeN_8_O_3_S_2_^+^ [M + H]^+^ requires 1310.6884, found 1310.6816 (–5.2 ppm).

#### **3Ce^iPr^(^t^BuNCS)**

To a solution of **1Ce^iPr^** (108 mg, 0.1 mmol) in DME (2 mL), ^t^BuNCS (34 μL, 0.3 mmol) was added and stirred for 2 h at 80 °C. The reaction mixture was cooled to room temperature and evaporated to dryness yielding a colourless powder (117 mg, 98%). ^1^H NMR (500 MHz, C_6_D_6_) *δ*_H_: –8.72 (3H, s), –8.44 (1H, s), –6.21 (3H, s), –4.08 (3H, s), –4.06 (9H, s), –3.74 (1H, s), –3.61 (3H, s), –2.35 (9H, s), –1.49 (1H, s), –0.10 (1H, s), 0.32 (1H, s), 1.50 (9H, s), 1.67 (9H, s), 1.98 (9H, s), 2.82 (1H, s), 3.25 (9H, s), 3.32 (3H, s), 3.51 (1H, s), 4.69 (1H, s), 6.08 (1H, s), 6.88 (1H, s), 8.34 (1H, s), 9.25–9.35 (9H, m), 9.82 (1H, s), 9.87 (1H, s), 14.04 (1H, s), 14.45 (3H, s), 17.40 (1H, s), 18.47 (9H, s). APPI^+^ C_65_H_96_CeN_7_O_3_S^+^ [M]^+^ requires 1194.6350, found 1194.6571 (+18.5 ppm). Elemental analysis C_65_H_96_CeN_7_O_3_S: C 65.29%, H 8.09%, N 8.20% calculated. C 65.42%, H 8.21%, N 7.59% found.

## Conflicts of interest

There are no conflicts to declare.

## Supplementary Material

Supplementary information

Crystal structure data
