# Supplementary material for: Selective and catalytic carbon dioxide and heteroallene activation mediated by cerium N-heterocyclic carbene complexes
Source: Chem Sci. 2018 Sep 10;9(42):8035–45. doi: 10.1039/c8sc03312a (PMC6262539; doi:10.1039/c8sc03312a)
Supplement: Supplementary file 1 [file SC-009-C8SC03312A-s001.pdf]

Electronic Supplementary Material (ESI) for Chemical Science.
This journal is © The Royal Society of Chemistry 2018

Selective and catalytic carbon dioxide and heteroallene activation mediated by cerium N-heterocyclic carbene complexes

Polly L. Arnold,[a,*] Ryan W. F. Kerr,[a,b] Catherine Weetman,[a] Scott R. Docherty,[a] Julia Rieb,[a,c] Kai Wang,[a] Christian Jandl,[a,c] Max W. McMullon,[a] Alexander Pöthig,[c,*] Fritz E. Kühn,[c,*] and Andrew D. Smith[b,*]

[a] *EaStCHEM School of Chemistry, University of Edinburgh, The King's Buildings, Edinburgh, EH9 3FJ, U.K.*
[b] *EaStCHEM School of Chemistry, University of St. Andrews, North Haugh, St. Andrews, KY16 9ST U.K.*
[c] *Molecular Catalysis, Faculty of Chemistry and Catalysis Research Center, Technical University Munich, Lichtenbergstr. 4, 85748 Garching bei München, Germany.*

Contents

1.1 Additional experiments ... 2
1.1.1 Reaction to target Sm(II)(O(*o*-C_6H_2-$^tBu_2$-2,6-CN(C_2H_2)N^{i}Pr)$_2$ ... 2
1.1.2 Reaction to target Ce(III)(O(*o*-C_6H_2-$^tBu_2$-2,6-CN(C_2H_2)N^{i}Pr)(N($SiMe_3$)$_2$)$_2$ ... 2
1.1.3 Reaction to target Ce(III)(O(*o*-C_6H_2-$^tBu_2$-2,6-CN(C_2H_2)N^{i}Pr)Cl_2 ... 2
1.1.4 Reaction to target Ce(III)(O(*o*-C_6H_2-$^tBu_2$-2,6-CN(C_2H_2)N^{i}Pr)$_2$Cl ... 2
1.1.5 Reaction of Ce(III)(O(*o*-C_6H_2-$^tBu_2$-2,6-CN(C_2H_2)N^{i}Pr)$_3$ and CS_2 ... 2
1.1.6 Reaction of Ce(III)(O(*o*-C_6H_2-$^tBu_2$-2,6-CN(C_2H_2)N^{i}Pr)$_3$ and cyclohexylallene ... 3
1.1.7 Reaction of Ce(III)(O(*o*-C_6H_2-$^tBu_2$-2,6-CN(C_2H_2)N^{i}Pr)$_3$ and ethylphenylketene ... 3
1.2 Catalytic formation of propylene carbonate ... 3
1.2.1 Using 2CeL^{iPr} ... 3
1.2.2 Using 2CeL^{Mes} ... 3
1.3 Mono ortho-ligand complex synthesis ... 4
1.3.1 4Ce^{Me} [Ce(O(*o*-C_6H_2-$^tBu_2$-2,6-CN(C_2H_2)NMe))(N^{i}Pr$_2$)Br.LiBr(THF)]$_2$ ... 4
1.3.2 4Ce^{iPr} [Ce(O(*o*-C_6H_2-$^tBu_2$-2,6-CN(C_2H_2)NiPr))(N^{i}Pr$_2$)Br.LiBr(THF)]$_2$ ... 4
1.3.3 5Ce^{Me} [Li(THF)Ce_3(O(*o*-C_6H_2-$^tBu_2$-2,6-CN(CHC)NMe))$_3$(N^{i}Pr$_2$)$_5$(THF)$_2$]$_2$ ... 5
1.4 Tris-para-ligand complex synthesis ... 5
1.4.1 [*p*-H_2L^{Mes}][Br] [HO(*p*-C_6H_2-$^tBu_2$-2,6-CHN(C_2H_4)NMes][Br] ... 5
1.4.2 [*p*-H_2L^{Mes}][PF_6] [HO(*p*-C_6H_2-$^tBu_2$-2,6-CHN(C_2H_4)NMes][PF_6] ... 6
1.5 Reactions to target 7Ln^{Mes}: ... 6
1.5.1 6Na^{Mes} [Na(O(*p*-C_6H_2-$^tBu_2$-2,6-CN(C_2H_4)NMes)]$_n$ ... 6
1.5.2 6K^{Mes} [K(O(*p*-C_6H_2-$^tBu_2$-2,6-CN(C_2H_4)NMes)(THF)$_2$]$_n$ ... 6
1.5.3 7Y^{Mes}·3KCl Y(O(*p*-C_6H_2-$^tBu_2$-2,6-CN(C_2H_4)NMes·3KCl)$_3$(THF)$_2$ ... 7
1.5.4 3Ce^{Mes}·3HCl Ce(O(*p*-C_6H_2-$^tBu_2$-2,6-CN(C_2H_4)NMes·3HCl)$_3$(THF)$_2$ ... 7
1.6 Crystallographic details ... 9
1.7 References ... 15

1.1 Additional experiments

1.1.1 Reaction to target Sm(II)(O(*o*-C_6H_2-$^tBu_2$-2,6-CN(C_2H_2)N^{i}Pr)$_2$

Using a modification of general procedure 1 – To a suspension of **[*o*-H_2L^{iPr}][Br]** (276 mg, 0.75 mmol) in DME (7.5 mL) was added KN($SiMe_3$)$_2$ (300 mg, 1.5 mmol) and the resulting mixture was stirred for 5 minutes at room temperature. SmI_2(thf)$_2$ (205 mg, 0.38 mmol) was added, and the resulting mixture instantly turned blue and was allowed to stir at room temperature for 2 hours by which time the solution was dark yellow. Volatiles were removed under reduced pressure, the crude product was extracted three times with hexane and the combined filtrates were concentrated to saturation, and cooled to –30 °C overnight. The resulting suspension was glass-fibre filtered and the solid collected and dried under vacuum to give Sm(III)(O(*o*-C_6H_2-$^tBu_2$-2,6-CN(C_2H_2)N^{i}Pr)$_3$ (**$1Sm^{iPr}$**) (40 %, 327 mg, 0.3 mmol).

1.1.2 Reaction to target Ce(III)(O(*o*-C_6H_2-$^tBu_2$-2,6-CN(C_2H_2)N^{i}Pr)(N($SiMe_3$)$_2$)$_2$

To a suspension of $CeCl_3$(thf)$_{1.15}$ (329 mg, 1 mmol), in THF (10 mL), was added solid KN($SiMe_3$)$_2$ (798 mg, 4 mmol) and the resulting slurry allowed to stir for 24 hours at room temperature. To the bright yellow reaction mixture was **[*o*-H_2L^{iPr}][Br]** (368 mg, 1 mmol) and stirred for a further 18 hours at room temperature. The reaction mixture was centrifuged, glass-fibre filtered and concentrated under reduced pressure to give an inseparable mixture of Ce(L^{iPr})$_3$ **$1Ce^{iPr}$** and potassium cerium "ate" salts that were not further investigated.

1.1.3 Reaction to target Ce(III)(O(*o*-C_6H_2-$^tBu_2$-2,6-CN(C_2H_2)N^{i}Pr)Cl_2

Using a modification of general procedure 1 – 3-(3,5-di-tert-butyl-2-hydroxyphenyl)-1-isopropyl-1*H*-imidazol-3-ium bromide **[*o*-H_2L^{iPr}][Br]** (394 mg, 1 mmol), KN($SiMe_3$)$_2$ (399 mg, 2 mmol), $CeCl_3$(thf)$_{1.15}$ (329 mg, 1 mmol) and DME (10 mL) gave after recrystallization **$1Ce^{iPr}$** as an impure yellow solid (215 mg, 0.2 mmol, 20 % based on empirical formula Ce(L^{iPr})$_3$).

1.1.4 Reaction to target Ce(III)(O(*o*-C_6H_2-$^tBu_2$-2,6-CN(C_2H_2)N^{i}Pr)$_2$Cl

Using a modification of general procedure 1 – 3-(3,5-di-tert-butyl-2-hydroxyphenyl)-1-isopropyl-1*H*-imidazol-3-ium bromide **[*o*-H_2L^{iPr}][Br]** (788 mg, 2 mmol), KN($SiMe_3$)$_2$ (798 mg, 4 mmol), $CeCl_3$(thf)$_{1.15}$ (329 mg, 1 mmol) and DME (10 mL) gave after recrystallization **$1Ce^{iPr}$** as an impure yellow solid (484 mg, 0.45 mmol, 45 % based on empirical formula Ce(L^{iPr})$_3$).

1.1.5 Reaction of Ce(III)(O(*o*-C_6H_2-$^tBu_2$-2,6-CN(C_2H_2)N^{i}Pr)$_3$ and CS_2

To a solution of Ce(L^{iPr})$_3$ **$1Ce^{iPr}$** (27 mg, 0.025 mmol) in C_6D_6 (0.6 mL) was added CS_2 (19 mg, 0.25 mmol) in a Youngs tap NMR tube. The solution was analysed by ^1H and ^{13}C NMR after 24 hours at room temperature showing only starting material. The reaction was heated to 80 °C for 48 hours and reanalysed by ^1H and ^{13}C NMR spectroscopy which confirmed that only starting materials were present.

1.1.6 Reaction of Ce(III)(O(*o*-C_6H_2-$^tBu_2$-2,6-CN(C_2H_2)N^{i}Pr)$_3$ and cyclohexylallene

To a solution of Ce(L^{iPr})$_3$ **1Ce^{iPr}** (27 mg, 0.025 mmol) in C_6D_6 (0.6 mL) was added cyclohexylallene (9 mg, 0.075 mmol) in a Youngs tap NMR tube. The solution was analysed by ^{1}H NMR after 24 hours at room temperature showing only starting material. The reaction was heated to 80 °C for 48 hours and reanalysed by ^{1}H NMR and only starting material was present.

1.1.7 Reaction of Ce(III)(O(*o*-C_6H_2-$^tBu_2$-2,6-CN(C_2H_2)N^{i}Pr)$_3$ and ethylphenylketene

To a solution of Ce(L^{iPr})$_3$ **1Ce^{iPr}** (109 mg, 0.1 mmol) in C_6H_6 (2 mL) was added ethylphenylketene[1] (14 mg, 0.1 mmol) in a capped vial and stirred for 5 minutes at RT. The solution was analysed by ^{1}H NMR spectroscopy which showed that all starting material was consumed and multiple products had formed which could not be isolated.

The reaction was also carried out at lower temperature (–20 °C), in different solvents (THF, DME) and at different ketene loadings (0.05 mmol, 0.3 mmol) with no obvious improvement in product distribution.

Ethylphenylketene was synthesised using a previously reported procedure by Smith *et al.*[1]

1.2 Catalytic formation of propylene carbonate

1.2.1 Using 2CeL^{iPr}

To a Youngs tap NMR tube charged with propylene oxide (68 µL, 1 mmol) in THF (0.6 mL) and C_6D_6 (2 drops) was added **2Ce^{iPr}** (12 mg, 0.01 mmol). The reaction mixture was freeze-pump-thaw degassed three times then exposed to an atmosphere of CO_2. The reaction was then heated to 80 °C for 7 days while monitoring by ^{1}H NMR spectroscopy at 24h intervals; no changes were observed.

1.2.2 Using 2CeL^{Mes}

To a Youngs tap NMR tube charged with propylene oxide (68 µL, 1 mmol) in THF (0.6 mL) and C_6D_6 (2 drops) was added **2Ce^{Mes}** (14 mg, 0.01 mmol). The reaction mixture was freeze-pump-thaw degassed three times then exposed to an atmosphere of CO_2. The reaction was then heated to 80 °C for 7 days while monitoring by ^{1}H NMR spectroscopy, yielding propylene carbonate (22%, 0.22 mmol) in accordance to literature values[2]; **^{1}H NMR** (500 MHz, C_6D_6) δ_H 0.50 (3H, d, *J* 6.4, $CH_aH_bCHCH_3$), 2.83 (1H, t, 7.6, $CH_aH_bCHCH_3$), 3.26 (1H, t, 7.6, $CH_aH_bCHCH_3$), 3.60 (1H, sext, *J* 6.6, $CH_aH_bCHCH_3$).**^{13}C{^{1}H} NMR** (126 MHz, C_6D_6) δ_C: 19.0 (CH_3), 70.7 (CH_2), 73.5 (CH_3), 154.6 (OC(O)O).

1.3 Mono ortho-ligand complexes

1.3.1 4Ce^{Me} [Ce(O(*o*-C_6H_2-$^tBu_2$-2,6-CN(C_2H_2)NMe))(N^iPr_2)Br.LiBr(THF)]$_2$

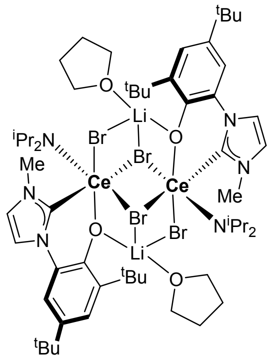

4Ce^{Me}

To a benzene suspension of **[*o*-H_2L^{Me}][Br]** (102 mg, 0.277 mmol, 1 mL) was added Ce(N^iPr_2)$_4$Li(THF) (200 mg, 0.323 mmol) slowly with occasional shaking, upon which a black suspension with a yellow glint was obtained. After standing for 5 h, a colourless precipitate had formed which was removed by centrifugation and the benzene supernatant decanted, and the solid extracted with 1 mL of benzene. The benzene solutions were combined in a vial and allowed to evaporate slowly. After 4 days, bright yellow crystals suitable for X-ray diffraction were isolated, washed with benzene (2x 0.5 mL) and hexane (2 x 0.5 mL) and allowed to dry at ambient pressure to yield the title compound **4Ce^{Me}** as a bright yellow solid (50 mg, 0.0327 mmol, 20% based on Ce, 47% based on Br). **^{1}H NMR** (500 MHz, C_6D_6): δ_H: –15.40 (6H, s, 2x N-C*H*$_3$), –9.87 (18H, s, 2x C(C*H*$_3$)$_3$), –1.37 (18H, s, 2x C(C*H*$_3$)$_3$), 1.80 (2H, s, 2x C*H*), 2.26 (2H, s, 2x C*H*), 2.76 (2H, s, 2x C*H*), 3.96 (2H, s, 2x C*H*), 4.65 (12H, br. s, 2x CH(C*H*$_3$)$_2$), 6.80 (8H, s, 4x THF-C*H*$_2$), 7.95 (2H, s, 2x C*H*), 9.41 (12H, br. s, 2x CH(C*H*$_3$)$_2$), 12.21 (8H, br. s, 4x THF-OC*H*$_2$). **^{7}Li NMR** (194.4 MHz, C_6D_6) δ_{Li}: 36.19. **Elemental Analysis** $C_{56}H_{96}Br_4Ce_2Li_2N_6O_4$: C 43.93%, H 6.32%, N 5.49% calculated. Found: C 43.88%, H 6.08%, N 5.40% found. **Evans method**: 1.7 mg in 0.65 mL of benzene, observed shift: 16.3 Hz (0.0326 ppm) -> μ = 5.1 μ_B.

Note: **5Ce^{Me}** was often observed to form in trace quantities in reactions to target **4Ce^{Me}** (identified by ^{7}Li NMR spectroscopy).

1.3.2 4Ce^{iPr} [Ce(O(*o*-C_6H_2-$^tBu_2$-2,6-CN(C_2H_2)NiPr))(N^iPr_2)Br.LiBr(THF)]$_2$

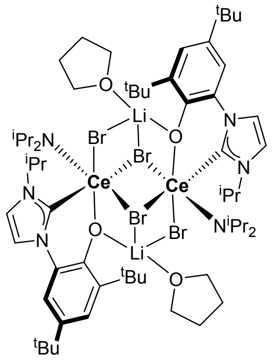

4Ce^{iPr}

Tetrabutylammonium bromide (78.0 mg, 0.242 mmol) and **[*o*-H_2L^{Me}][Br]** (95.7 mg, 0.242 mmol) was dissolved/suspended in 1.5 mL of benzene and Ce(N^iPr_2)$_4$Li(THF) (150 mg, 0.242 mmol) was added with occasional shaking. After stirring for 5 h, the white precipitate was removed by centrifugation and the yellow solution was stored in a semi-closed vial for crystallisation. After 3 days, bright yellow crystals suitable for X-ray diffraction were obtained and washed with benzene (0.5 mL) then hexane (2 x 1 mL) and stored openly for 1 day to remove residual solvent to yield the title compound **4Ce^{iPr}** as a bright yellow solid 72.8 mg (0.046 mmol, 38%) as yellow crystals. **^{1}H NMR** (500 MHz, C_6D_6) δ_H: –30.48 (2H, s, 2x NHC-NC*H*(CH$_3$)), –10.00 (18H, s, 2x C(C*H*$_3$)$_3$), –8.46 (6H, s, 2x NHC-NCH(C*H*$_3$)), –7.53 (6H, s, 2x NHC-NCH(C*H*$_3$)), –1.74 (4H, s, 2x C*H*(CH$_3$)$_2$), –1.27 (18H, s, 2x C(C*H*$_3$)$_3$), 1.85 (2H, s, 2x C*H*), 2.57 (2H, s, 2x C*H*), 4.06 (2H, s, 2x C*H*), 5.40 (12H, br. s, 2x CH(C*H*$_3$)$_2$), 6.15 (8H, s, 4x THF-C*H*$_2$), 8.09 (2H, s, 2x C*H*), 10.68 (12H, br. s., 2x CH(C*H*$_3$)$_2$), 12.68 (8H, br. s, 4x THF-OC*H*$_2$). **^{7}Li** (194.4 MHz, C_6D_6) δ_{Li}: 37.56. **Elemental Analysis** $C_{60}H_{104}Br_4Ce_2Li_2N_6O_4$: C 45.40 %, H 6.60, N, 5.29 calculated. C 45.33%, H 6.61%, N 5.38% found. **Magnetic moment (Evan's solution method):** 1.5 mg in 0.65 mL of benzene, observed shift: 10.6 Hz (0.0212 ppm) -> μ = 4.6 μ_B.

1.3.3 5Ce^{Me} [Li(THF)Ce$_3$(O(*o*-C$_6$H$_2$-^{t}Bu$_2$-2,6-CN(CHC)NMe))$_3$(N^{i}Pr$_2$)$_5$(THF)$_2$]$_2$

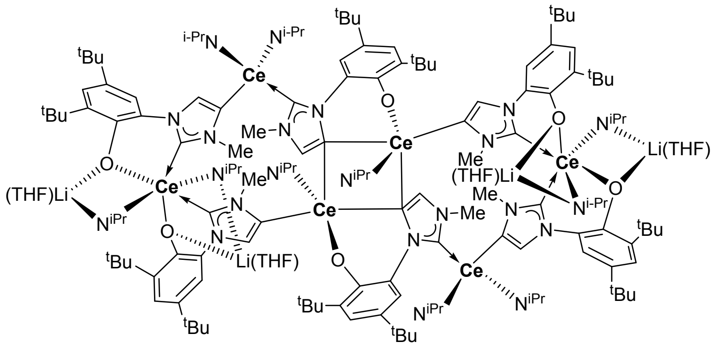

5Ce^{Me} (N^{iPr} = N(^{i}Pr)$_2$)

An NMR tube was loaded with **[*o*-H$_2$L^{Me}][Br]** (15.0 mg, 40.8 µmol) and Ce(^{i}Pr$_2$N)$_4$Li(THF) (32 mg, 51.6 µmol) and ca. 0.5 mL of C$_6$D$_6$. After ca. 7 days, the formation of bright orange crystals, which were suitable for X-ray diffraction, was observed as **5Ce^{Me}** but upon repeated attempts could never isolated on a larger scale**.** **^{7}Li NMR** (194.4 MHz, C$_6$D$_6$) δ_{Li}: 38.01.

1.4 Tris-para-ligand complex

1.4.1 [*p*-H$_2$L^{Mes}][Br] [HO(*p*-C$_6$H$_2$-^{t}Bu$_2$-2,6-CHN(C$_2$H$_4$)NMes][Br]

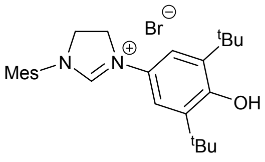

[*p*-H$_2$L^{Mes}][Br]

An ampoule was charged with 4-bromo-2,4,6-tri-tert-butyl-2,5-cyclohexadien-1-one (6.82 g, 20 mmol), *N*-mesitylimidazoline (7.4285g, 40 mmol) and ethylene glycol (1.1 ml, 20 mmol). The mixture was heated to 135 °C for 8 h. After cooling to room temperature, 50 mL of HBr aqueous solution (3 M) was added and the mixture stirred for 2 hours. Chloroform was added to extract (2 x 30 ml) and the organic layer was separated and dried over anhydrous Na$_2$SO$_4$. Removal of volatiles by rotary evaporator to afford a brown oil, a colourless precipitate was isolated after recrystallisation from a 50/50 mix of CHCl$_3$/THF at –30 °C (15 %, 3 mmol, 1.41 g). **^{1}H NMR** (500 MHz, CDCl$_3$) δ_H: 1.46 (18H, s, Ar^1(2,6)C(C*H*$_3$)$_3$), 2.28 (3H, s, Ar^2(4)C*H*$_3$), 2.38 (6H, s, 6H, Ar^2(2,6)C*H*$_3$)), 4.51 (2H, t, *J* 12.5, NC*H*$_2$CH$_2$N), 4.84 (2H, t, *J* 12.5, NCH$_2$C*H*$_2$N), 6.93 (2H, s, Ar^2(3,5)*H*), 7.30 (2H, s, Ar^1(3,5)*H*), 9.55 (1H, s, NC*H*N). **^{13}C{^{1}H} NMR** (126 MHz, CDCl$_3$) δ_C: 18.4, 21.0, 30.1, 34.8, 50.8, 51.7, 76.9, 117.0, 127.5, 129.9, 130.7, 135.1, 137.8, 140.2, 153.7, 155.1. **Elemental Analysis** C$_{26}$H$_{37}$N$_2$OBr: C 65.95%, H 7.88%, N 5.92% calculated. C 65.76%, H 7.98%, N 5.84% found.

1.4.2 [*p*-H$_2$L^{Mes}][PF$_6$] [HO(*p*-C$_6$H$_2$-^{t}Bu$_2$-2,6-CHN(C$_2$H$_4$)NMes][PF$_6$]

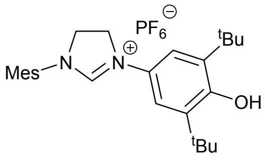

[*p*-H$_2$L^{Mes}][PF$_6$]

To a stirred solution of **[*p*-H$_2$L^{Mes}][Br]** (1 g**,** 2.1 mmol) in KPF$_6$ (aq., 10 M, 50 mL), was added a 50/50 mixture of Et$_2$O and DCM (50 mL). After vigorous stirring for two hours, the organic layer was separated and dried over MgSO$_4$, filtered and concentrated under reduced pressure. The title product was purified though a flash

silica column by using 9:1 DCM/ acetone ratio (94%, 1.06g, 1.97 mmol). **^{1}H NMR** (500 MHz, d_8-THF) δ_H: 1.48 (18H, s, $Ar^1(2,6)C(CH_3)_3$), 1.66 (3H, s, $Ar^2(4)CH_3$), 2.31 (6H, s, 6H, $Ar^2(2,6)CH_3$)), 4.32 (2H, t, *J* 10.3, NCH_2CH_2N), 4.70 (2H, t, *J* 10.3, NCH_2CH_2N), 6.53 (2H, s, $Ar^2(3,5)H$), 6.94 (2H, s, $Ar^1(3,5)H$), 8.69 (1H, s, NC*H*N). **^{13}C{^{1}H} NMR** (126 MHz d_8-THF) δ_C: 16.9, 20.2, 29.5, 34.8, 50.5, 51.1, 110.0, 117.4, 129.6, 131.5, 135.8, 139.2, 140.0, 154.1, 155.6. **^{19}F NMR** (471 MHz, d_8-THF) δ_F: –72.68 (d, $J_{F\text{-}P}$ 712.1). **^{31}P NMR** (202 MHz, d_8-THF) δ_P: -143.87 (s, $J_{P\text{-}F}$ 710.7).

1.5 Reactions to target 7Ln^{Mes}:

1.5.1 6Na^{Mes} [Na(O(*p*-C_6H_2-$^tBu_2$-2,6-CN(C_2H_4)NMes)]$_n$

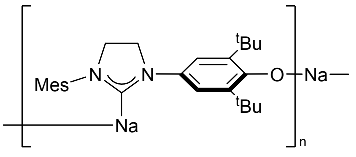

6Na^{Mes}

To a THF suspension of **[*p*-H_2L^{Mes}][Br]** (30 mg, 0.063 mmol), 2 equivalents of NaN" (23 mg, 0.126 mmol) was added at –30 °C and then slowly allowed to warm to room temperature. Precipitates were removed by centrifugation and volatiles were removed from the supernatant under reduced pressure to yield the title compound in quantitative yields. Samples of **6Na^{Mes}** for synthetic procedures were always made *in situ*, but single crystals can be obtained by slow evaporation of a concentrated THF solution. **^{1}H NMR** (500 MHz, d_8-THF) δ_H: 1.41 (18H, s, $Ar^1(2,6)C(CH_3)_3$), 2.20 (6H, s, 6H, $Ar^2(2,6)CH_3$)), 2.24 (3H, s, $Ar^2(4)CH_3$), 3.61 (2H, m, NCH_2CH_2N), 3.90 (2H, m, NCH_2CH_2N), 6.85 (2H, s, $Ar^2(3,5)H$), 7.07 (2H, s, $Ar^1(3,5)H$). **^{13}C NMR** (126 MHz, d_8-THF) δ_C: 18.5, 21.1, 25.2, 30.8, 49.9, 50.9, 115.4, 125.9, 129.4, 135.5, 136.1, 137.1, 141.3, 167.7, 237.2.

1.5.2 6K^{Mes} [K(O(*p*-C_6H_2-$^tBu_2$-2,6-CN(C_2H_4)NMes)(THF)$_2$]$_n$

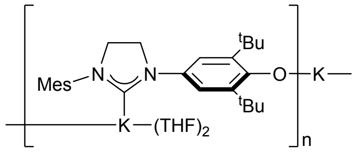

6K^{Mes}

To a THF suspension of **[*p*-H_2L^{Mes}][Cl]** (30 mg, 0.063 mmol), 2 equivalents of KN" (25 mg, 0.126 mmol) was added at –30 °C and then slowly warmed to room temperature. Precipitate were removed by centrifugation and then volatiles were removed from the supernatant under reduced pressure to yield the title compound in quantitative yields. Single crystals were obtained through slow diffusion of hexane into a concentrated THF solution (63%, 25 mg, 0.04 mmol). **^{1}H NMR** (500 MHz, NC_5D_5) δ_H: 1.42 (18H, s, $Ar^1(2,6)C(CH_3)_3$), 2.21 (6H, s, 6H, $Ar^2(2,6)CH_3$)), 2.24 (3H, s, $Ar^2(4)CH_3$), 3.61 (2H, m, NCH_2CH_2N), 3.90 (2H, m, NCH_2CH_2N), 6.85 (2H, s, $Ar^2(3,5)H$), 7.03 (2H, s, $Ar^1(3,5)H$). **^{13}C NMR** (126 MHz, NC_5D_5) δ_C: 18.5, 21.1, 30.7, 35.9, 50.3, 50.0, 116.1, 125.0, 129.5, 135.6, 136.2, 137.1, 141.2, 168.6, 237.0. **Elemental Analysis** $C_{34}H_{52}KN_2O_3$: C 70.91%, H 9.10%, N 4.86% calculated. C 70.58%, H 9.27%, N 4.73% found.

1.5.3 7Y^{Mes}·3KCl Y(O(*p*-C_6H_2-$^tBu_2$-2,6-CN(C_2H_4)NMes·3KCl)$_3$(THF)$_2$

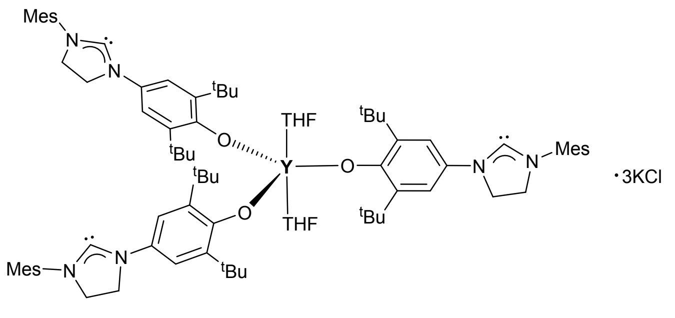

$7Y^{Mes}\cdot 3KCl$

A THF solution of 6 eq. of KN″ (150 mg, 0.75 mmol, 10 mL) was added to **[p-H_2L^{Mes}][PF_6]** (199 mg, 0.37 mmol) at –30 °C and stirred for 30 minutes to give a pale-yellow solution. A THF (15 mL) solution of $YCl_3(THF)_2$ (41 mg, 0.12 mmol) was then added at -30 °C and the mixture allowed to warm to room temperature over 4h with stirring, to afford a pale-yellow solution and a colourless precipitate. The solution was centrifuged to remove the salt by-products, and the volatiles removed from the supernatant under reduced pressure to yield a solid material of **$7Y^{Mes}\cdot 3KCl$** which was washed with hexane and dried (56%, 88 mg, 0.21 mmol). **^{1}H NMR** (500 MHz, NC_5D_5) δ_H: 1.91 (54H, s, 3x $Ar^1(2,6)C(CH_3)_3$), 2.18 (9H, br. s, 3x $Ar^2(4)CH_3$), 2.26 (18H, br. s, 3x $Ar^2(2,6)CH_3$)), 3.68 (12H, m, 3x NCH_2CH_2N), 6.90 (6H, s, 3x $Ar^2(3,5)H$), 7.86 (6H, s, 3x $Ar^1(3,5)H$). **^{13}C NMR** (151 MHz, NC_5D_5) δ_C: 18.8, 21.4, 31.2, 36.3, 50.9, 51.3, 117.1, 125.3, 136.6, 136.8, 136.8, 138.5, 138.5, 138.5, 236.0. **Elemental Analysis** $C_{86}H_{119}Cl_3K_3N_6O_5Y$: C 63.39%, H 7.36%, N 5.16% calculated. C 62.65%, H 7.70%, N 6.15% found.

1.5.4 $3Ce^{Mes}\cdot 3HCl$ $Ce(O(p\text{-}C_6H_2\text{-}^tBu_2\text{-}2,6\text{-}CN(C_2H_4)NMes\cdot 3HCl)_3(THF)_2$

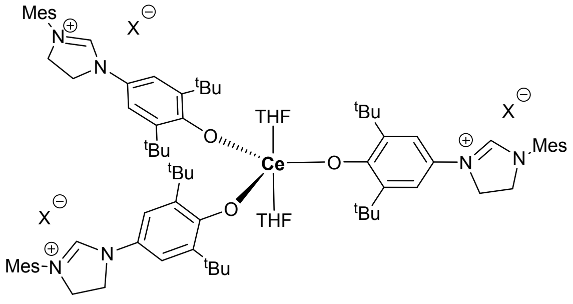

$7Ce^{Mes}\cdot 3HX$

A THF solution of 6 eq. of NaN″ (138 mg, 0.75 mmol, 10 mL) was added to **[p-H_2L^{Mes}][Br]** (175 mg, 0.37 mmol) at –30 °C and stirred for 30 minutes to give a pale-yellow solution. A THF (15 mL) solution of $CeCl_3(THF)_{1.1}$ (39 mg, 0.12 mmol) was then added at -30 °C and the mixture allowed to warm to room temperature while stirring for 4 hours, to give a pale-yellow solution with a colourless precipitate. The solution was centrifuged to remove the salt by-products, and the volatiles removed from the supernatant *in vacuo* to yield a solid material of **$7Ce^{Mes}\cdot 3HX$** which was washed with hexane and dried (30%, 52 mg, 0.08 mmol). Note: The final product degrades rapidly into an unknown yellow/brown powder within several hours of isolation preventing further characterisation. **^{1}H NMR** (500 MHz, C_6D_6) δ_H: 0.22 (54H, s, 3x $Ar^1(2,6)C(CH_3)_3$), 1.69 (9H, s, 3x $Ar^2(4)CH_3$), 1.93 (18H, s, 3x $Ar^2(2,6)CH_3$)), 3.69 (6H, m, 3x NCH_2CH_2N), 4.05 (6H, m, 3x NCH_2CH_2N), 6.62 (6H, s, 3x $Ar^1(3,5)H$), 6.93 (6H, s, 3x

$Ar^2(3,5)H$), 7.33 (3H, s, 3x NC*H*N). **^{13}C NMR** (151 MHz, d_8-THF) δ_C: 14.2, 17.4, 20.8, 23.1, 30.2, 32.0, 35.5, 50.5, 51.6, 116.6, 129.9, 132.1, 136.0, 138.0, 139.8, 152.6.

.

1.6 Crystallographic details

The molecular structures of **1Ce^{iPr}·C$_6$H$_{14}$**, **1Ce^{Mes}·C$_6$H$_6$**, **Ce^{iPr}·C$_7$H$_8$·0.5(C$_6$H$_{14}$)**, **2Ce^{tBu}·C$_6$H$_{14}$**, **3Ce^{iPr}(^{t}BuNCS)**, **4Ce^{Me}·C$_6$H$_6$**, **4Ce^{iPr}**, **5Ce^{Me}·2(C$_6$H$_6$)**, **6Na^{Mes}**, **6K^{Mes}·2(OC$_4$H$_8$)**, **1Ce^{iPr}.HBr·0.5(C$_6$H$_6$)** and **1Eu^{iPr}.HBr·3(C$_7$H$_8$)** were solved using SHELXT[3] and least-square refined using SHELXL[4] in Olex2[5]. Hydrogen atoms were treated by constrained refinement with the exception of **6Na^{Mes}** which was mixed refinement. **1Ce^{iPr}** – C(181)/C(182)/C(183) were positionally disordered over two positions and were split into two parts and refined with an occupancy ratio of 0.4:0.6. In addition, the thermal parameters were restrained using the RIGU command, and the bond angles were restrained through the use of the SADI command. C(381)/C(382)/C(383) were positionally disordered over two positions and were split into two parts and refined with an occupancy ratio of 0.4:0.6. In addition, the thermal parameters were set to be similar by use of the RIGU command, and the bond angles were restrained through the use of the SADI command. **1Ce^{Mes}** – C(381)/C(383)/C(385) were positionally disordered over two positions and were split into two parts and refined with an occupancy ratio of 0.3:0.7. In addition, the thermal parameters were constrained to be equal for C(382)/C(383) by use of the EADP command, the quaternary carbon atom of a positionally disordered *tert*-butyl group. **2Ce^{iPr}** – C(1S)/C(2S)/C(3S)/C(4S)/C(5S)/C(6S)/C(7S) (toluene lattice solvent) was disordered and constrained using the DELU and SIMU commands. C(8S)/C(9S)/C(10S) (Hexane lattice solvent) was disordered and constrained using the DELU and SIMU commands. The squeeze function[6] was used to remove 180 electrons from solvent accessible voids, which equates to approximately 3 toluene molecules. **2Ce^{tBu}** – C(1S)/C(2S)/C(3S)/C(4S)/C(5S)/C(6S)/C(7S) (hexane lattice solvent) was highly disordered and constrained using the SADI, DELU, SIMU and ISOR commands. The squeeze function[6] was used to remove 206 electrons from solvent accessible voids, which equates to approximately 4 hexane molecules. **3Ce^{iPr}(^{t}BuNCS) –** C(180)/C(181)/C(182)/C(183) were positionally disordered over two positions and were split into two parts and fixed to 0.5:0.5 occupancy. In addition, the thermal parameters were set to be similar by use of the SIMU command, and the bond angles were restrained through the use of the SADI command. Two solvent accessible voids of 80 electrons were excluded from the lattice using the olex solvent mask feature, which was believed to be highly disordered heptane. **4Ce^{Me}** – The THF ligand, diisopropyl amide ligand, and one *tert*-butyl group were positionally disordered and a split layer refinement with the sum of occupancies set to 1 was applied. In addition, thermal parameters were constrained by use of the SAME, SIMU and DELU command. **5Ce^{Me}** – The THF ligands, *tert*-butyl groups and benzene lattice solvent molecule were positionally disordered and a split layer refinement with the sum of occupancies set to 1 was applied. In addition, thermal parameters were constrained by use of the SAME, SADI, SIMU and RIGU command. The squeeze function[6] was used to remove 150 electrons from solvent accessible voids, which equates to approximately 4 benzene molecules. **6Ka^{Mes}** – O(1S)/C(1S)/C(2S)/C(3S)/C(4S) and O(2S)/C(5S)/C(6S)/C(7S)/C(8S) (coordinated THF) was disordered and constrained using the DELU, SIMU and RIGU commands. **1Ce^{iPr}.HBr –** Single crystals were once grown from a concentrated solution of **1Ce^{iPr}** in benzene stored at room temperature overnight. R^1 = 10.55%, the structure is included for connectivity only. In addition 34 electrons were excluded from the lattice using the olex solvent mask feature, which was believed to be highly disordered benzene. **1Eu^{iPr}.HBr –** Single crystals were once grown from a concentrated solution of **1Eu^{iPr}** in toluene stored at room

temperature overnight. C(183) is positionally disordered over two positions, and was spilt into C(183) and C(184) and refined with an occupany ratio of 0.6:0.4.

Table S1. Crystallographic data for **1Ce^{iPr}**, **1Ce^{Mes}**, **2Ce^{iPr}**, and **2Ce^{tBu}**.

Complex	[Ce(L^{iPr})$_3$] (**1Ce^{iPr}**)	[Ce(L^{Mes})$_3$] (**1Ce^{Mes}**)	[Ce(L^{iPr}.CO$_2$)$_3$] (**2Ce^{iPr}**)	[Ce(L^{tBu}.CO$_2$)$_3$] (**2Ce^{tBu}**)
Local code	p17049_077_mono_sq	p17079	po17034_sq	p17042_tri2_sq
Chemical formula	$0.5(C_{60}H_{87}CeN_6O_3)\cdot 0.5(C_6H_{14})$	$C_{78}H_{99}CeN_6O_3\cdot C_6H_6$	$2(C_{63}H_{87}CeN_6O_9)\cdot 2(C_7H_8)\cdot C_6H_{14}$	$C_{66}H_{93}CeN_6O_9\cdot C_6H_{14}$
M_r	583.32	1386.85	2761.45	1386.69
Crystal system, space group	Monoclinic, $P2_1/c$	Monoclinic, $P2_1/c$	Triclinic, $P^{-}1$	Triclinic, $P^{-}1$
Temperature (K)	293	120	120	120
a, *b*, *c* (Å)	18.8567 (3), 20.0714 (4), 19.2011 (4)	12.6458 (2), 24.6116 (4), 24.4523 (4)	15.9574 (7), 17.7857 (4), 17.8098 (5)	17.1931 (6), 18.0941 (5), 18.1420 (5)
b (°)	102.741 (2)	93.800 (1)	62.686 (2), 69.389 (3), 77.518 (3)	60.078 (3), 63.808 (3), 83.013 (3)
V (Å^3)	7088.3 (2)	7593.6 (2)	4194.6 (3)	4353.9 (3)
Z	8	4	1	2
Radiation type	Mo *K*a	Mo *K*a	Cu *K*a	Mo *K*a
m (mm^{-1})	0.69	0.65	4.60	0.57
Crystal size (mm)	0.39 × 0.21 × 0.18	0.55 × 0.13 × 0.11	0.25 × 0.2 × 0.14	0.33 × 0.21 × 0.10
Diffractometer	Xcalibur, Eos	Xcalibur, Eos	SuperNova, Dual, Cu at zero, Atlas	SuperNova, Dual, Cu at zero, Atlas
Absorption correction	Analytical *CrysAlis PRO* 1.171.38.46 (Rigaku Oxford Diffraction, 2015) Analytical numeric absorption correction using a multifaceted crystal model based on expressions derived by R.C. Clark & J.S. Reid. (Clark, R. C. & Reid, J. S. (1995). Acta Cryst. A51, 887-897) Empirical absorption correction using spherical harmonics, implemented in SCALE3 ABSPACK scaling algorithm.	Analytical *CrysAlis PRO* 1.171.38.46 (Rigaku Oxford Diffraction, 2015) Analytical numeric absorption correction using a multifaceted crystal model based on expressions derived by R.C. Clark & J.S. Reid. (Clark, R. C. & Reid, J. S. (1995). Acta Cryst. A51, 887-897) Empirical absorption correction using spherical harmonics, implemented in SCALE3 ABSPACK scaling algorithm.	Multi-scan *SADABS*	Gaussian *CrysAlis PRO* 1.171.38.46 (Rigaku Oxford Diffraction, 2015) Numerical absorption correction based on gaussian integration over a multifaceted crystal model Empirical absorption correction using spherical harmonics, implemented in SCALE3 ABSPACK scaling algorithm.
T_{min}, T_{max}	0.922, 0.960	0.948, 0.984	0.322, 0.472	0.406, 1.000
No. of measured, independent and observed [*I* > 2s(*I*)] reflections	73360, 16030, 11616	80694, 18515, 13872	84368, 17387, 14813	118520, 19977, 16930
R_{int}	0.075	0.059	0.075	0.093
(sin θ/λ)$_{max}$ (Å^{-1})	0.649	0.689	0.631	0.650

$R[F^2 > 2s(F^2)]$, $wR(F^2)$, S	0.098, 0.226, 1.04	0.046, 0.144, 0.93	0.097, 0.272, 0.95	0.057, 0.147, 1.04
No. of reflections	16030	18515	17387	19977
No. of parameters	773	899	807	822
No. of restraints	172		81	78
H-atom treatment	H-atom parameters constrained	H-atom parameters constrained	H-atom parameters constrained	H-atom parameters constrained
$\Delta)_{max}$, $\Delta)_{min}$ (e Å^{-3})	8.47, -4.41	0.89, -0.46	2.89, -1.60	2.40, -1.07

Table S2. Crystallographic data for **$3Ce^{iPr}(^tBuNCS)_2$**, **$4Ce^{Me}$**, **$4Ce^{iPr}$**, and **$5Ce^{Me}$**

Complex	$[L^{iPr}Ce(L^{iPr}.^tBuNCS)_2]$ (**$3Ce^{iPr}(^tBuNCS)_2$**)	$(Ce_2Br_4L^{Me}(^iPr_2N)_2Li_2(THF)_2)$ (**$4Ce^{Me}$**)	$(Ce_2Br_4L^{iPr}(^iPr_2N)_2Li_2(THF)_2)$ (**$4Ce^{iPr}$**)	$Li_2[Ce_3(L^{Me})_3(N\{^iPr\}_2)_5](THF)_2$ (**$5Ce^{Me}$**)
Local code	p18052_mono	p17026	po17005	rieju40
Chemical formula	$C_{70}H_{105}CeN_8O_3S_2$	$C_{56}H_{94}Br_4Ce_2Li_2N_6O_4{\cdot}C_6H_6$	$C_{60}H_{102}Br_4Ce_2Li_2N_6O_4$	$C_{184}H_{316}Ce_6Li_4N_{22}O_{10}{\cdot}2(C_6H_6)$
M_r	1310.85	1607.20	1585.20	4021.30
Crystal system, space group	Monoclinic, $P2_1/c$	Monoclinic, $P2_1/n$	Triclinic, P^-1	Triclinic, P^-1
Temperature (K)	293	120	120	100
a, b, c (Å)	16.4910 (3), 25.6698 (4), 18.9736 (4)	14.0830 (3), 17.4883 (2), 15.0209 (2)	11.2409 (5), 11.4341 (6), 15.3361 (9)	14.2898 (16), 17.937 (2), 21.837 (2)
b (°)	100.574 (2)	99.155 (2)	88.292 (5), 77.751 (4), 64.883 (5)	100.428 (2), 94.826 (3), 90.276 (3)
V (Å^3)	7895.5 (3)	3652.34 (10)	1740.08 (18)	5484.1 (10)
Z	4	2	1	1
Radiation type	Mo *K*a	Mo *K*a	Mo *K*a	Mo *K*a
m (mm^{-1})	0.67	3.46	3.63	1.27
Crystal size (mm)	0.33 × 0.27 × 0.15	0.58 × 0.41 × 0.17	0.52 × 0.17 × 0.07	0.67 × 0.29 × 0.23
Diffractometer	Xcalibur, Eos	Xcalibur, Eos	SuperNova, Dual, Cu at zero, Atlas	Bruker Photon CMOS
Absorption correction	Analytical *CrysAlis PRO* 1.171.38.46 (Rigaku Oxford Diffraction, 2015) Analytical numeric absorption correction using a multifaceted crystal model based on expressions derived by R.C. Clark & J.S. Reid. (Clark, R. C. & Reid, J. S. (1995). Acta Cryst. A51, 887-897) Empirical absorption correction using spherical harmonics, implemented in	Multi-scan *CrysAlis PRO* 1.171.38.46 (Rigaku Oxford Diffraction, 2015) Empirical absorption correction using spherical harmonics, implemented in SCALE3 ABSPACK scaling algorithm.	Gaussian *CrysAlis PRO* 1.171.38.46 (Rigaku Oxford Diffraction, 2015) Numerical absorption correction based on gaussian integration over a multifaceted crystal model Empirical absorption correction using spherical harmonics, implemented in SCALE3 ABSPACK scaling algorithm.	Multi-scan *SADABS* 2014/5, Bruker, 2014

	SCALE3 ABSPACK scaling algorithm.			
T_{min}, T_{max}	0.957, 0.978	0.496, 1.000	0.149, 1.000	0.623, 0.745
No. of measured, independent and observed [$I > 2s(I)$] reflections	144651, 13441, 9734	77453, 7464, 6117	35056, 7104, 6262	155945, 20083, 15865
R_{int}	0.137	0.045	0.044	0.049
(sin q/l)$_{max}$ (Å^{-1})	0.588	0.625	0.625	0.602
$R[F^2 > 2s(F^2)]$, $wR(F^2)$, S	0.054, 0.144, 1.00	0.035, 0.088, 1.07	0.026, 0.056, 1.03	0.052, 0.132, 1.06
No. of reflections	13441	7464	7104	20083
No. of parameters	827	526	364	1340
No. of restraints	625	537	0	981
H-atom treatment	H-atom parameters constrained	H-atom parameters constrained	H-atom parameters constrained	H-atom parameters constrained
Δ)$_{max}$, Δ)$_{min}$ (e Å^{-3})	1.87, -0.97	1.32, -0.78	0.87, -0.65	3.88, -2.41

Table S3. Crystallographic data for **6Na^{Mes}**, **6K^{Mes}**, **1Ce^{iPr}.HBr** and **1Eu^{iPr}.HBr**

Complex	[(Na(*p*-L^{Mes})]$_n$ (**6Na^{Mes}**)	[(K(*p*-L^{Mes})]$_n$ (**6K^{Mes}**)	[(L^{iPr})$_2$Ce(L^{iPr}.HBr)] (**1Ce^{iPr}.HBr**)	[(L^{iPr})$_2$Eu(L^{iPr}.HBr)] (**1Eu^{iPr}.HBr**)
Local code	p16052	p15081_lowercut	p15125bmaxp1	po4046_refinalized
Chemical formula	$C_{26}H_{35}N_2NaO$	$C_{34}H_{51}KN_2O_3$	$C_{60}H_{87}BrCeN_6O_3 \cdot C_3H_3$	$C_{60}H_{88}BrEuN_6O_3 \cdot 3(C_7H_8)$
M_r	414.55	574.86	1199.43	1449.63
Crystal system, space group	Triclinic, $P\bar{}1$	Monoclinic, $P2_1/n$	Triclinic, $P\bar{}1$	Triclinic, $P\bar{}1$
Temperature (K)	293	293	293	120
a, *b*, *c* (Å)	8.6969 (7), 12.2469 (10), 12.7912 (9)	11.5409 (3), 16.2694 (5), 17.8385 (6)	12.0853 (18), 16.255 (3), 19.608 (3)	13.6772 (3), 17.0307 (4), 18.4376 (4)
a, b, g (°)	73.287 (7), 79.755 (6), 69.269 (7)	90, 91.020 (3), 90	68.047 (18), 80.606 (14), 68.922 (18)	81.2919 (19), 70.526 (2), 69.910 (2)
V (Å^3)	1215.95 (18)	3348.88 (18)	3331.7 (12)	3799.37 (17)
Z	2	4	2	2
Radiation type	Mo *K*a	Mo *K*a	Mo *K*a	Cu *K*a
m (mm^{-1})	0.08	0.19	1.33	6.90
Crystal size (mm)	0.24 × 0.24 × 0.10	0.72 × 0.12 × 0.09	0.53 × 0.16 × 0.07	0.21 × 0.09 × 0.07
Diffractometer	Xcalibur, Eos	Xcalibur, Eos	Xcalibur, Eos	SuperNova, Dual, Cu at zero, Atlas

Absorption correction	Analytical *CrysAlis PRO*, Agilent Technologies, Version 1.171.37.35 (release 13-08-2014 CrysAlis171 .NET) (compiled Aug 13 2014,18:06:01) Analytical numeric absorption correction using a multifaceted crystal model based on expressions derived by R.C. Clark & J.S. Reid. (Clark, R. C. & Reid, J. S. (1995). Acta Cryst. A51, 887-897) Empirical absorption correction using spherical harmonics, implemented in SCALE3 ABSPACK scaling algorithm.	Analytical *CrysAlis PRO*, Agilent Technologies, Version 1.171.37.35 (release 13-08-2014 CrysAlis171 .NET) (compiled Aug 13 2014,18:06:01) Analytical numeric absorption correction using a multifaceted crystal model based on expressions derived by R.C. Clark & J.S. Reid. (Clark, R. C. & Reid, J. S. (1995). Acta Cryst. A51, 887-897) Empirical absorption correction using spherical harmonics, implemented in SCALE3 ABSPACK scaling algorithm.	Multi-scan *CrysAlis PRO*, Agilent Technologies, Version 1.171.37.35 (release 13-08-2014 CrysAlis171 .NET) (compiled Aug 13 2014,18:06:01) Empirical absorption correction using spherical harmonics, implemented in SCALE3 ABSPACK scaling algorithm.	Gaussian *CrysAlis PRO*, Agilent Technologies, Version 1.171.37.34k (release 13-08-2014 CrysAlis171 .NET) (compiled Aug 13 2014,16:31:05) Numerical absorption correction based on gaussian integration over a multifaceted crystal model Empirical absorption correction using spherical harmonics, implemented in SCALE3 ABSPACK scaling algorithm.
T_{min}, T_{max}	0.951, 0.978	0.632, 0.918	0.631, 1.000	0.981, 0.994
No. of measured, independent and observed [I > 2s(I)] reflections	12583, 2543, 1843	59632, 5317, 3803	53040, 13608, 7284	94322, 15813, 14980
R_{int}	0.076	0.068	0.176	0.046
q_{max} (°)	20.8	24.1		
(sin q/l)$_{max}$ (Å^{-1})	0.500	0.575	0.625	0.630
R[F^2 > 2s(F^2)], wR(F^2), S	0.059, 0.164, 1.01	0.076, 0.217, 1.04	0.106, 0.315, 0.90	0.051, 0.150, 1.05
No. of reflections	2543	5317	13608	15813
No. of parameters	289	370	691	867
No. of restraints	0	140	30	0
H-atom treatment	H atoms treated by a mixture of independent and constrained refinement	H-atom parameters constrained	H-atom parameters constrained	H-atom parameters constrained
Δ)$_{max}$, Δ)$_{min}$ (e Å^{-3})	0.24, -0.17	0.76, -0.37	3.14, -1.57	0.76, -3.41

Computer programs: *CrysAlis PRO* 1.171.38.46 (Rigaku OD, 2015), *APEX* III Control Software (Bruker, 2015), *CrysAlis PRO*, Agilent Technologies, Version 1.171.37.35 (release 13-08-2014 CrysAlis171 .NET) (compiled Aug 13 2014, 18:06:01), Version 1.171.37.34k (release 13-08-2014 CrysAlis171 .NET) (compiled Aug 13 2014, 16:31:05), *SAINT* (Bruker, 2014), *SHELXT* (Sheldrick, 2015), OLEX2 (Dolomanov, 2009), olex2.solve (Bourhis *et al.*, 2015), *SHELXL2014* (Sheldrick, 2014), SHELXLE (Huebschle, 2011), *SHELXL2014* (Sheldrick, 2015), *SHELXL* (Sheldrick, 2015), Olex2 (Dolomanov *et al.*, 2009), *PLATON* (Spek, 2011), *enCIFer* (Allen, 2014).

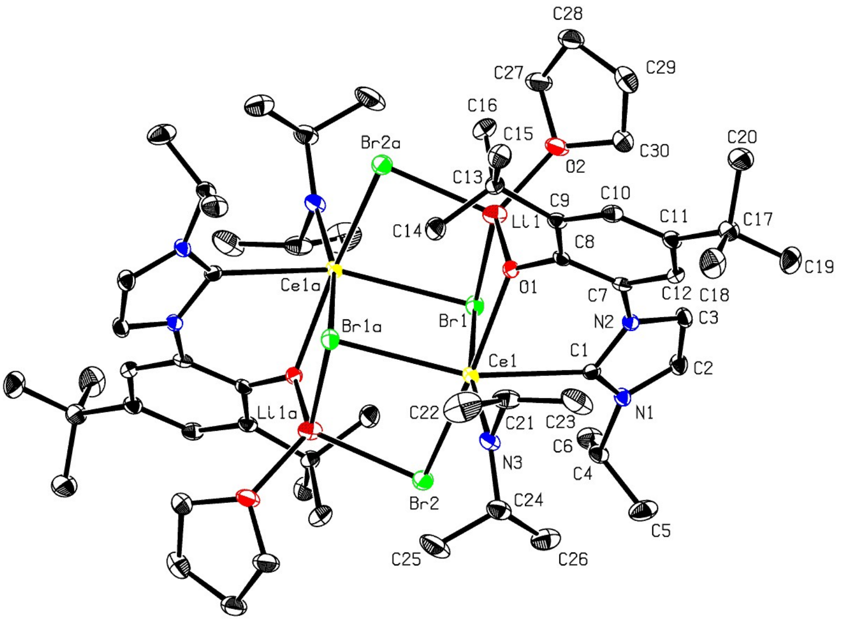

Figure S1: Molecular structure of $Ce_2Br_4L^{iPr}{}_2(N^iPr_2)_2Li_2(THF)_2$ **4Ce^{iPr}** in the solid state with ellipsoids at the 50% probability level. Hydrogen atoms are omitted for clarity. Selected distances (Å) and angles (°): Ce1—Br1 3.2802(5), Ce1—Br2 2.9738(4), Ce1—Br1a 3.0306(4), Ce1—O1 2.392(2), Ce1—N3 2.244(2), Ce1—C1 2.641(3), Li1—Br1 2.620(5), Li1—Br2 2.573(5), Li1—O1 1.916(5), Li1—O2 1.952(6), Ce1···C21 2.982(3), Ce1···H21{C21} 2.62, Ce1···C24 3.500(3), Ce1—Br1—Ce1a 97.32(1), Br1—Ce1—Br1a 80.27(1), Ce1—Br1—Li1 74.19(12), Ce1—Br2a—Li1a 92.43(11), Ce1—O1—Li1 112.75(17), C1—Ce1—N3 94.37(9), Ce1—O1—C8 120.88(14), Ce1—C1—N1 132.5(2), Ce1—C1—N2 120.31(15), Ce1—C1—centroid(C1,N1,C2,C3,N2) 162.57, Li1—O1—C8 120.3(2).

Crystals of **4Ce^{iPr}** were obtained by slow evaporation of a solution in benzene. **4Ce^{iPr}** crystallises in the triclinic spacegroup $P\bar{1}$ as a dimer with an internal centre of inversion (see Figure S1). The compound consists of two Ce(III) ions, two ligands **L1^{iPr}**, four bromides, two diisopropylamide ligands and two lithium ions, which are each coordinated by a THF molecule. Ce(III) is coordinated in a distorted octahedral fashion. Two bromides exhibit a μ^3-bridging mode between two Ce(III) and one Li(I) with one Ce—Br distance significantly longer than the other (3.2802(5) Å vs. 2.9738(4) Å). The other bromides exhibit a μ^2-bridging mode between Ce(III) and Li(I), same as the aryloxide moiety of the ligand **L1^{iPr}**. The NHC moiety coordinates to Ce(III) in the normal carbene mode with a Ce—$C_{carbene}$ distance of 2.641(3) Å. The coordination shows both in-plane displacement (Ce1—C1—N1 = 132.5(2)° vs. Ce1—C1—N2 120.31(15)°) and out-of-plane displacement (Ce1—C1—centroid(NHC) = 162.57°). Notably, the diisopropylamide ligand is significantly tilted, illustrated by the difference of the Ce—C21 and Ce—C24 distances (2.982(3) Å vs. 3.500(3) Å). Although hydrogen positions cannot be exactly located, this indicates an interaction between Ce(III) and the H atom on C21 with an approximate Ce—H distance of 2.62 Å, based on the calculated H position which is consistent with the orientation of the respective isopropyl group.

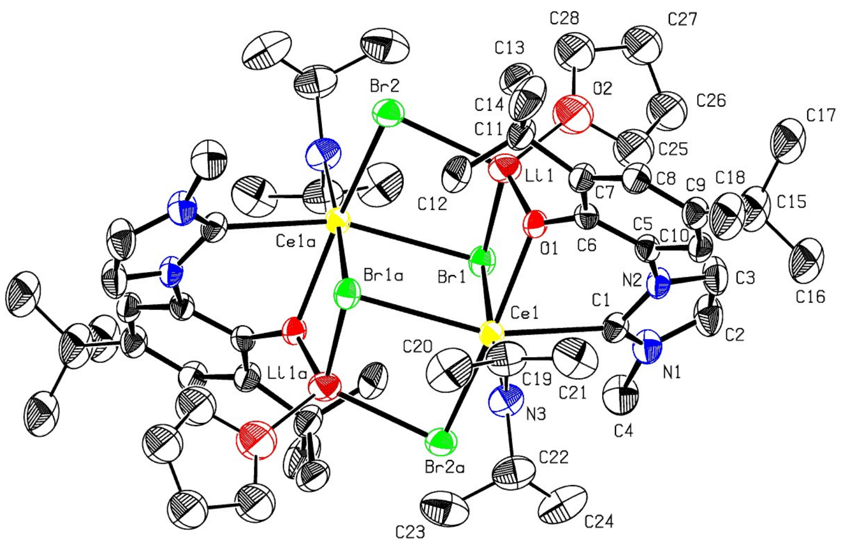

Figure S2: Molecular structure of $Ce_2Br_4L^{Me}{}_2(N^iPr_2)_2Li_2(THF)_2$ **4Ce^{Me}** in the solid state with ellipsoids at the 50% probability level. Hydrogen atoms and co-crystallized benzene are omitted for clarity. Disordered groups are indicated by thin bonds. Selected distances (Å) and angles (°): Ce1—Br1 3.2427(5), Ce1—Br2 2.9732(5), Ce1—Br1a 3.0085(5), Ce1—O1 2.396(3), Ce1—N3 2.235(5), Ce1—C1 2.639(4), Li1—Br1 2.627(8), Li1—Br2 2.586(8), Li1—O1 1.908(8), Li1—O2 1.947(15), Ce1···C19 2.976(7), Ce1···H19{C19} 2.61, Ce1···C22 3.494(6), Ce1—Br1—Ce1a 98.73(1), Br1—Ce1—Br1a 81.27(1), Ce1—Br1—Li1 72.66(16), Ce1—Br2a—Li1a 88.92(17), Ce1—O1—Li1 108.9(2), C1—Ce1—N3 100.14(18), Ce1—O1—C6 118.2(2), Ce1—C1—N1 135.7(3), Ce1—C1—N2 119.0(3), Ce1—C1—centroid(C1,N1,C2,C3,N2) 165.45, Li1—O1—C6 130.3(3).

Crystals of **4Ce^{Me}** were obtained by slow evaporation of a solution in benzene. The compound crystallizes in the monoclinic spacegroup $P2_1/n$ as a dimer with an internal centre of inversion (see Figure S2) and with half an equivalent of co-crystallized benzene. The molecular structure is analogous to **$Ce_2Br_4L1^{iPr}{}_2(^iPr_2N)_2Li_2(THF)_2$**. Bond lengths are also comparable (e.g. Ce—$C_{carbene}$ = 2.639(4)), only the angles between coordinating atoms around the central cluster of the structure show small differences due to the different wingtip. The degree of distortion of the Ce-NHC coordination is higher in-plane (Ce1—C1—N1 = 135.7(3)° vs. Ce1—C1—N2 119.0(3)°) and lower out-of-plane (Ce1—C1—centroid(NHC) = 165.45°) as compared to the isopropyl derivative. Similar to the isopropyl derivative, a tilted diisopropylamide ligand is observed, indicating an interaction of Ce(III) with the H atom on C19. Due to the disorder of this ligand, however, this is less certain than in the isopropyl derivative.

1.7 References

1. J. Douglas James, G. Churchill, M. Z. Slawin Alexandra, J. Fox David and D. Smith Andrew, *Chem. Eur. J.*, 2015, **21**, 16354-16358.
2. H. Zhou, W.-Z. Zhang, C.-H. Liu, J.-P. Qu and X.-B. Lu, *J. Org. Chem.*, 2008, **73**, 8039-8044.
3. G. Sheldrick, *Acta Cryst. A*, 2015, **71**, 3-8.
4. G. Sheldrick, *Acta Cryst. C*, 2015, **71**, 3-8.
5. O. V. Dolomanov, L. J. Bourhis, R. J. Gildea, J. A. K. Howard and H. Puschmann, *J. Appl. Crystallogr.*, 2009, **42**, 339-341.
6. A. Spek, *Acta Cryst. C.* 2015, **71**, 9-18.
